# SARS-CoV2 Nsp1 is a metal-dependent DNA and RNA endonuclease

**DOI:** 10.1007/s10534-024-00596-z

**Published:** 2024-03-28

**Authors:** Bruno A. Salgueiro, Margarida Saramago, Mark D. Tully, Federico Issoglio, Sara T. N. Silva, Ana C. F. Paiva, Cecília M. Arraiano, Pedro M. Matias, Rute G. Matos, Elin Moe, Célia V. Romão

**Affiliations:** 1 ITQB-NOVA, Instituto de Tecnologia Química e Biológica António Xavier, Universidade Nova de Lisboa, Avenida da República, 2780-157 Oeiras, Portugal; 2 ESRF, European Synchrotron Radiation Facility, 71, avenue des Martyrs CS 40220, 38043 Grenoble Cedex 9, France; 3 iBET, Instituto de Biologia Experimental e Tecnológica, Apartado 12, 2780-901 Oeiras, Portugal; 4 Department of Chemistry, UiT, the Arctic University of Norway, Tromsø, Norway

**Keywords:** Coronavirus, Nuclease, Manganese, Magnesium, Biophysics, SAXS

## Abstract

Over recent years, we have been living under a pandemic, caused by the rapid spread of the severe acute respiratory syndrome coronavirus 2 (SARS-CoV2). One of the major virulence factors of Coronaviruses is the Non-structural protein 1 (Nsp1), known to suppress the host cells protein translation machinery, allowing the virus to produce its own proteins, propagate and invade new cells. To unveil the molecular mechanisms of SARS-CoV2 Nsp1, we have addressed its biochemical and biophysical properties in the presence of calcium, magnesium and manganese. Our findings indicate that the protein in solution is a monomer and binds to both manganese and calcium, with high affinity. Surprisingly, our results show that SARS-CoV2 Nsp1 alone displays metal-dependent endonucleolytic activity towards both RNA and DNA, regardless of the presence of host ribosome. These results show Nsp1 as new nuclease within the coronavirus family. Furthermore, the Nsp1 double variant R124A/K125A presents no nuclease activity for RNA, although it retains activity for DNA, suggesting distinct binding sites for DNA and RNA. Thus, we present for the first time, evidence that the activities of Nsp1 are modulated by the presence of different metals, which are proposed to play an important role during viral infection. This research contributes significantly to our understanding of the mechanisms of action of Coronaviruses.

**Supplementary Information:**

The online version contains supplementary material available at 10.1007/s10534-024-00596-z.

## Introduction

Coronaviruses (CoVs) are enveloped positive-sense single-stranded RNA viruses belonging to the *Coronaviridae* family, which comprises four genera: alpha-, beta-, gamma-, and delta-coronavirus. Among these, beta-CoVs are known to cause respiratory diseases, namely the Severe Acute Respiratory Syndrome (SARS), the Middle East Respiratory Syndrome (MERS-CoV) and the more recent SARS-CoV2 which led to the emergence of the disease now widely known as COVID-19 (Ksiazek et al. 2003; Zaki et al. 2012; de Wit et al. 2016; Lai et al. 2020).

The 30 kb RNA genome of SARS-CoV2 encodes 29 viral proteins, including Nsp1, a small *ca.* 20 kDa protein unique to alpha/beta CoV (HASÖKSÜZ et al. 2020; Gordon et al. 2020b). Previous studies have shown that Nsp1 interferes with the host innate immune system, suppressing part of its antiviral response (Züst et al. 2007; Yuan et al. 2020).

Different functions have been described for Nsp1 from alpha and beta-CoVs. To date, it has demonstrated an ability to inhibit translation, promoting mRNA degradation, with the possible exception of TGEV Nsp1 (Kamitani et al. 2006, 2009; Narayanan et al. 2008; Wang et al. 2010; Huang et al. 2011; Lokugamage et al. 2012; Terada et al. 2017; Yuan et al. 2021; Abaeva et al. 2023; Tardivat et al. 2023; Shehata and Parker 2023). Furthermore, Nsp1 directly binds to the small ribosomal subunit (40S) during translation initiation (Simeoni et al. 2021; Burke et al. 2021). This interaction was also recently demonstrated for the MERS-CoV Nsp1 (Devarkar et al. 2023). However, this function has not yet been observed for the Transmissible gastroenteritis virus (TGEV) Nsp1 (Yuan et al. 2021). It has been shown that Nsp1 promotes RNA degradation in the 5´UTR of mRNA, however the degradation was proposed to require the interaction of the mRNA with the ribosome (Abaeva et al. 2023; Tardivat et al. 2023; Shehata and Parker 2023). Moreover, it has been reported that Nsp1^SARS−CoV2^ blocks mRNA export by binding to NXF1 of the NXF1-NXT1 mRNA export receptor (Zhang et al. 2021). Additionally, it was observed that Nsp1^SARS−CoV^ associates with the nucleoporin Nup93, displacing it from the nuclear pore complex (Gomez et al. 2019). Several studies have been performed to elucidate how Nsp1 mediates endonucleolytic cleavages of both host and viral mRNAs. It has been proposed that SL1 hairpin at the 5´ end plays a crucial role in protecting mRNA from Nsp1-mediated degradation (Sosnowski et al. 2022; Abaeva et al. 2023; Tardivat et al. 2023). However, an open question remains regarding the mechanism by which Nsp1 degrades host mRNA and not the viral mRNA.

Structurally, Nsp1 is composed of two domains: an N-terminal domain (NTD, residues 1–128) that comprises alpha helices and beta-strands, a C-terminal domain (CTD, residues 148–180) with two helices and a connecting loop (residues 129–147) linking both domains. The protein structure of the NTD of Nsp1^SARS−CoV2^ has been determined through NMR and X-ray crystallography (PDB codes:7K3N; 8A4Y; 8AYS; 8A55; 8AZ8; 8CRF; 8CRK; 8CRM; 7ZQ4) (Semper et al. 2021; Zhao et al. 2021; Ma et al. 2022, 2023; Borsatto et al. 2022). The structure of the C-terminal has been determined in complex with the 40S small subunit of the ribosome by CryoEM (PDB: 6ZLW; 6G5H;6Y0G) (Shi et al. 2020; Thoms et al. 2020; Schubert et al. 2020; Yuan et al. 2021). The NMR structure of the Nsp1^SARS−CoV2^ full length protein (PDB code: 8AOU) was also recently reported (Wang et al. 2023).

The diverse functions observed for Nsp1^SARS−CoV2^ suggest a multifunctional role within the cell during infections. However, several questions remain unanswered, hindering a complete understanding of its involvement in the mechanisms of host cell infection. We have correlated the function of Nsp1^SARS−CoV2^ with the importance of oligo-elements such as calcium (Ca^2+^), magnesium (Mg^2+^) and manganese (Mn^2+^) in the SARS-CoV2 viral infection cycle (da Silva and Williams 2001; Chaturvedi and Shrivastava 2005; Zhou et al. 2009; Oliveira et al. 2014; Chen 2018; Wang et al. 2018; Chang-Graham et al. 2019; Iotti et al. 2020; Berlansky et al. 2022; Sun et al. 2023). Studies have reported that calcium concentrations outside the cells can reach up to 10 mM (Zhou et al. 2009; Chang-Graham et al. 2019). However, within the endoplasmic reticulum and the cytosol, this concentration remains in the hundreds of µM and nM ranges, respectively, tightly regulated by transporters or pumps (Zhou et al. 2009; Chang-Graham et al. 2019). It has also been reported that many viruses disrupt the normal calcium balance of cells by increasing citric acid levels while simultaneously decreasing endoplasmic reticulum and mitochondrial salt concentrations upon infection (Zhou et al. 2009; Oliveira et al. 2014; Chang-Graham et al. 2019; Berlansky et al. 2022). This rise in cytosolic calcium concentration activates or accelerates several enzyme-dependent processes, increasing ATP production to meet the substantial energy demand for viral replication. Thus, viruses influence cytosolic calcium levels, accelerating or inducing apoptotic cell death to facilitate virus liberation and propagation (Chaturvedi and Shrivastava 2005; Zhou et al. 2009; Chang-Graham et al. 2019).

Magnesium plays a role in the development of an immature and adaptable immune system, typically maintained at a concentration of 1 mM in mammalian cells (da Silva and Williams 2001; Iotti et al. 2020). It has been shown that a low level of magnesium in human cells, associated with disease related to hypomagnesemia, correlates with a higher risk of developing COVID-19 (Iotti et al. 2020).

Manganese ions are required for many physiological processes, but its cellular levels are generally kept low, ranging from 0.072 to 0.27 µM (Aschner and Aschner 2005; Wang et al. 2018). Studies indicate that after a viral infection, cytosolic Mn^2+^ levels increase to 5.8–6.8 µM due to the release of manganese stored in the mitochondria (Wang et al. 2018; Sun et al. 2023). Manganese is also required in the host defense against viruses, enhancing sensor sensitivity (Wang et al. 2018; Sun et al. 2023). Moreover, it is important to consider the role of elements in the DNA replication machinery during infectious and inflammatory processes associated with viral infection (Chaturvedi and Shrivastava 2005; Li et al. 2022).

Nsp1^SARS−CoV2^ has been extensively studied, and its interaction with ribosomes has been reported (Simeoni et al. 2021; Burke et al. 2021; Abaeva et al. 2023; Tardivat et al. 2023; Shehata and Parker 2023). Also it has been shown that Nsp1 mediates endonucleolytic cleavages of both host and viral mRNA in the 5´UTR, but this degradation requires the mRNA interaction with the ribosome (Simeoni et al. 2021; Burke et al. 2021; Abaeva et al. 2023; Tardivat et al. 2023; Shehata and Parker 2023). However, the mechanism of action of this viral protein requires further investigation. Therefore, we have performed a comprehensive biochemical and biophysical characterization, revealing that this protein alone, regardless of the presence of host ribosome, can process both DNA and RNA molecules, acting as a metal-dependent endonuclease. Considering the crucial role that different metals play during viral infection (Chaturvedi and Shrivastava 2005; Li et al. 2022), we hypothesize that metals, namely calcium, magnesium, or manganese may modulate its endonucleolytic activity. Notably, this study demonstrated for the first time a connection between different metal ions and the enzymatic function of Nsp1^SARS−CoV2^. This breakthrough represents a significant advance to fully understand the multifunctional roles of Nsp1 in the cell and moving towards controlling the Coronavirus infections.

## Results

### Effect of metals in the secondary structure composition

**Fig. 1:**
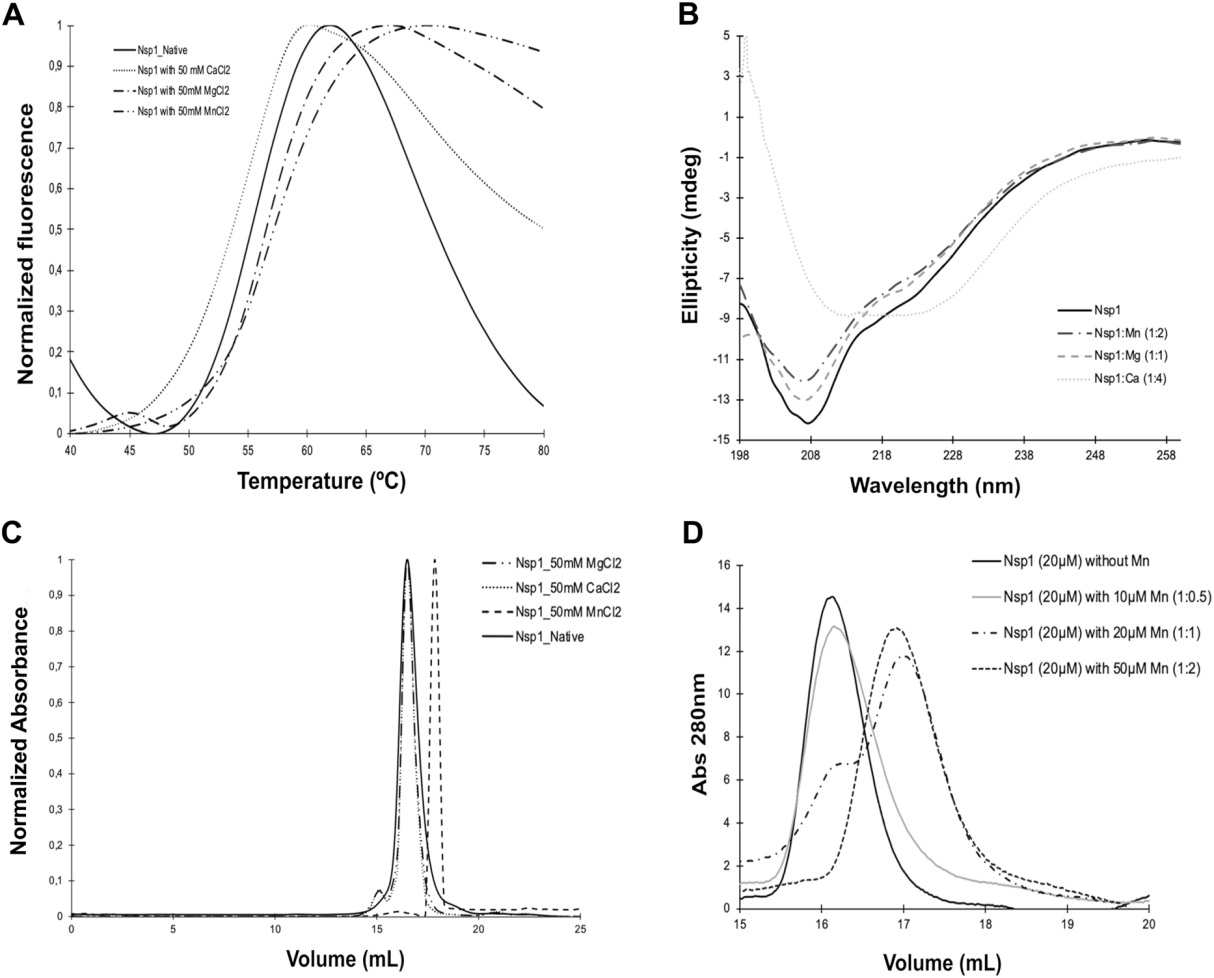
Thermal shift assay (TSA), Circular Dichroism (CD) spectroscopy and Size exclusion chromatography (SEC) of Nsp1^SARS−CoV2^. **A** TSA of native Nsp1^SARS−CoV2^ protein (0.13 mg mL^−1^) without metals (black line), with 50 mM of MgCl_2_ (-. -), CaCl_2_ (...) or MnCl_2_ (-.. -). **B** Secondary structure composition determined by CD spectroscopy of Nsp1^SARS−CoV2^ protein (0.1 mg mL^−1^) with and without different metals, Far-UV CD spectra of the protein without any metal (black line) in 20 mM sodium phosphate pH 6.5 with 1:4 equivalent of different CaCl_2_ (yellow lines), with 1:1 equivalents MgCl_2_ (green lines) and with 1:2 equivalents of MnCl_2_ (purple lines). **C** SEC of Nsp1 ^SARS−CoV2^ (1.0 mg mL^−1^) without any metal (control, black line), with 50 mM MgCl_2_ (-.. -), CaCl_2_ (...) or with MnCl_2_ (- – -). **D** SEC of Nsp1 ^SARS−CoV2^ with different ratios of Nsp1:MnCl_2_: protein without MnCl_2_ (control, black line), ratio 1:0.5 (gray line), ratio 1:1 (line – - -), and the ratio 1:2 (line -. -)

Nsp1 is considered one of the main virulence factors from SARS-CoVs, which counteracts part of the host response mechanism against a viral infection (Züst et al. 2007; Huang et al. 2011; Lokugamage et al. 2012; Yuan et al. 2020). Based on previous knowledge regarding the potential roles of different metals during virus infection and nucleic acids metabolism, three metals were selected for studies on their effect on the activity of Nsp1: calcium, manganese, and magnesium (Chaturvedi and Shrivastava 2005; Iotti et al. 2020; Li et al. 2022; Berlansky et al. 2022). A thermal shift assay was performed to understand how these metals affect the structural fold of Nsp1. The results indicate a slight increase in the melting temperature (T_M_) of Nsp1^SARS−CoV2^ in the presence of Mg^2+^ (∆T_M_ =  + 1ºC) and Mn^2+^ (∆T_M_ =  + 2ºC), while a decrease of the T_M_ was observed (∆T_M_ = -2ºC) in the case of Ca^2+^, compared with the native conditions (Fig. 1A). At temperatures above 65ºC, most curves exhibited a decrease in fluorescence signal (Fig. 1A). This has been previously described and may be linked to protein aggregation, as the reduced surface area limits dye binding (Niesen et al. 2007; Johnson et al. 2014; Samuel et al. 2021). Notably, Nsp1^SARS−CoV2^ under native conditions showed a more significant decrease compared to when the protein was in the presence of metals Ca, Mg and Mn, suggesting that these metals could prevent protein aggregation.

To gain a deeper understanding of the impact that these metals have on the stability of Nsp1, the secondary structure composition was assessed by Circular Dichroism (CD) (Fig. 1B). In the absence of metals, Nsp1^SARS−CoV2^ presents a high percentage of random coil, similar to what is reported for the homologous Nsp1^SARS−CoV^ (Brucz et al. 2007). The high percentage of random coil (*ca.* 45%) suggests that this protein is highly flexible, as observed in Nsp1^SARS−CoV^ which presents 39% random coil. This result is comparable to that obtained for the CTD of Nsp1^SARS−CoV2^ (Kumar et al. 2021), based on our results it is evident that the full-length Nsp1^SARS−CoV2^ has a higher secondary structure content than Nsp1^SARS−CoV^ (Brucz et al. 2007).

The impact of different metals on the overall structure was tested, and the addition of metals tends to reduce the secondary structure contents when compared with native Nps1 (Fig. 1B). An increase in calcium concentration, induces an increase of the negative ellipticities at 210–230 nm, reflecting a decrease in α-helical and β-sheet contents when compared with the native protein. On the other hand, the random coil content increases to *ca.* 62%, suggesting that Nsp1^SARS−CoV2^ adopts a more flexible conformation in the presence of calcium. In the presence of magnesium, the bands at 209 and 220 nm become more pronounced (Fig. 1B). Additionally, a decrease in the molar ellipticity, in the region of 205–230 nm is observed (Fig. 1B), indicating an increase in random coil content to *ca.* 64%, while the secondary structure contents (mostly β-sheet) decreases. The presence of manganese induces minor changes on its secondary structure, since the CD spectrum of the protein incubated with manganese presents only slightly differences compared with the spectrum for the native sample (Fig. 1B).

To deepen our knowledge of the interaction of Nsp1^SARS−CoV2^ with the different metals, and correlate it with its biological function, the interaction affinity and the thermodynamic parameters were determined by Isothermal Titration Calorimetry (ITC). Upon injection of the different metals (Ca^2+^; Mg^2+^; Mn^2+^) to Nsp1^SARS−CoV2^, heat is released or absorbed, with each subsequent injection, the change in heat generated decreases as the Nsp1^SARS−CoV2^ protein in the cell becomes saturated with the metal (Fig. S1). The addition of different metals to Nsp1^SARS−CoV2^ results in a spontaneous process. The results show that the reaction with calcium and manganese is exothermic, while the addition of magnesium is endothermic (Table 1). Moreover, the number of binding sites and dissociation constant for each metal were different (Table 1). Calcium and manganese presented higher affinity than for magnesium, *K*_*D*_* (Ca*^*2*+^*)* = 6.2 ± 0.9 nM and four binding sites, and *K*_*D*_* (Mn*^*2*+^*)* = 11.7 ± 3.2 nM and two binding sites (Table 1).

**Table 1:** The number of divalent metals per Nsp1 protein, apparent dissociation constant (*K*_*D*_), and thermodynamic parameters of Nsp1^SARS−CoV2^ interactions at 25 °C

Nsp1_SARS_CoV_2_	*K*_*D*_ (nM)	N^a^	*△H* kcal.mol^−1^	*△S* cal.mol^−1^.K^−1^	*△G* kcal.mol^−1b^
Nsp1—Ca^2+^	6.22 ± 0.9	~ 4 ± 0.2	− 8.07 ± 0.3	1.30	− 8.46
Nsp1—Mg^2+^	3300 ± 252.6	~ 1 ± 0.5	7.10 ± 0.9	49.00	− 7.51
Nsp1—Mn^2+^	11.70 ± 3.2	~ 2 ± 0.1	− 8.30 ± 1.1	8.40	− 10.80

^a^number of Nsp1 binding sites

^b^*ΔG* was calculated as *ΔH-TΔS*

To further complement these studies, the oligomeric state of Nsp1^SARS−CoV2^ was analyzed by Size-Exclusion Chromatography (SEC) in the presence of the different metals (Fig. 1C). The elution profile of Nsp1^SARS−CoV2^ in the native condition, incubated with Ca^2+^, or Mg^2+^, is identical, corresponding to a monomeric form (1.2 ± 0.2). However, the protein incubated with Mn^2+^, presented a different elution profile although it stills corresponds to a monomeric form (0.7 ± 0.04) (Fig. 1C). This may indicate that the presence of manganese promotes a conformational change with a lower hydrodynamic radius. The oligomeric form was assigned according to a calibration curve prepared with globular protein standards. Interestingly, the addition of two equivalents of manganese to Nsp1^SARS−CoV2^ leads to a complete shift from the one conformation to the other (Fig. 1D).

### Different conformations of Nsp1

**Fig. 2:**
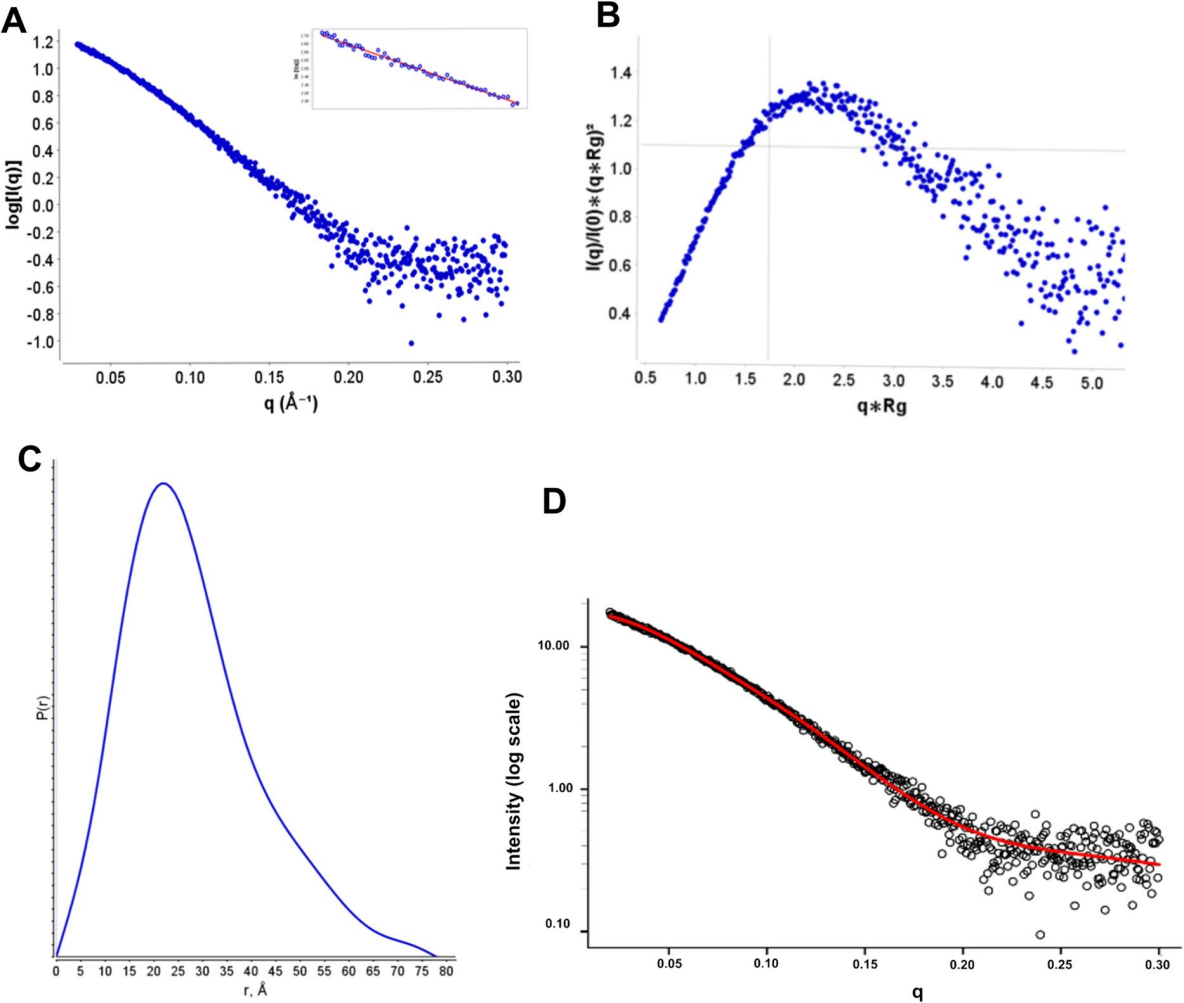
SAXS of Nsp1^SARS−CoV2^. **A** Log10 SAXS intensity versus scattering vector, *q*. The plotted range represents the positive only data within the specified *q*-range. The plot insert shows the straight line of the Guinier plot from which the R_g_ is derived. **B** Normalized Kratky plot. Plot demonstrates that Nsp1^SARS−CoV2^ is elongated (peak above the crosshairs) but also globular (peak falls below horizontal gray line). **C** Pair-distance, P(r), distribution function. Maximum dimension, D_max_, is the largest non-negative value that supports a smooth distribution function. **D** Computed profile built by MultiFoXS using a 5-state model (red line) and SAXS profile obtained with recombinant Nsp1 ^SARS_CoV2^ (black circles). (Color figure online)

To confirm the oligomeric state of the native protein as monomeric, Nsp1^SARS−CoV2^ was analysed using the structural technique, Small Angle X-ray Scattering (SAXS) that provides data on the size and shape of the protein in solution. The Guinier plots, derived from a linear fit to the scattering profile show that the solution is monodisperse (Fig. 2A) and give a radius of gyration (R_g_) of 22.64 Å (Table S1). Using the R_g_ the scattering profile can be transformed into a normalised Kratky plot. The gaussian peak from the Kratky plot indicates that the protein is folded (Fig. 2B). An indirect Fourier transform, of the scattering data will derive the pair distance distribution function [P(r)], a histogram of all the distances within the protein including the max dimension (D_max_), determined from the scattering profile curves using ScatterIV (Tully et al. 2021). The P(r) profile of Nsp1^SARS−CoV2^ showed a *D*_max_ of 78 Å (Fig. 2C, Table S1). It was also possible to determine a molecular mass of ~ 24 kDa for Nsp1^SARS−CoV2^ and compare it with the calculated theoretical molecular mass determined from the known sequences of 19.78 kDa, together this data suggests that Nsp1^SARS−CoV2^ is a monomer in its native form (Table S1).

These results indicated that the protein in the native state has an elongated globular shape, reflected in the lower elution volume from the SEC analysis (Fig. 1C). Since the elution profile is identical in the presence of either calcium or magnesium, it is suggested that under these conditions the protein presents a similar conformation as in the native state. However, in the presence of manganese, it acquires a globular shape, resulting a higher elution volume (Fig. 1C and D).

**Fig. 3:**
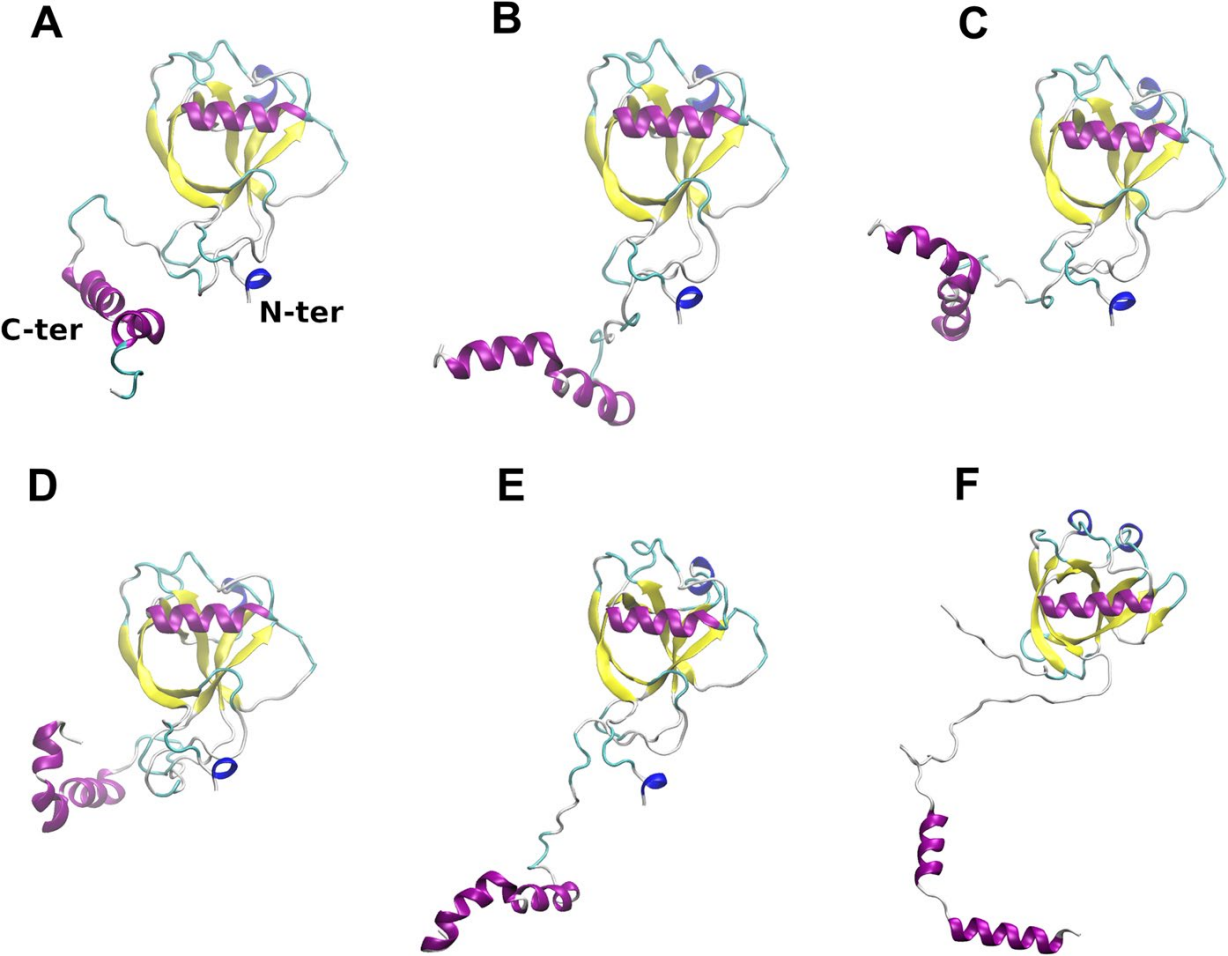
Conformations obtained with MultiFoXS and structural model of Nsp1^SARS−CoV2^. **A–E** Conformations obtained with MultiFoXS for a 5-state model, where the Rg values for each conformation are 22.2, 22.5, 21.6, 19.5 and 28.1. For the 1-state model the unique conformation obtained is the same as the first structure from the 5-state model (**A**). **F** Homology model obtained with MODELLER

Given the flexibility of the Nsp1^SARS−CoV2^ CTD, it is expected that a macroscopic observation such as a SAXS profile would present a heterogeneous ensemble of conformations. Therefore, to obtain a set of models that would agree with the SAXS results we used MultiFoXS (Schneidman-Duhovny et al. 2016). With this tool it is possible to evaluate different conformations by defining flexible residues in the protein, and consequently improving the fitting of the resulting models to the experimental SAXS profile provided (Fig. 2D). MultiFoXS was employed to model the missing residues in the crystallographic structure of Nsp1^SARS−CoV2^ (PDB code 7K7P; residues 1 to 9 and 128 to 180) (Clark et al. 2021) and find the conformations that best fit our data. We obtained 5 different conformations whose contribution explains our experimental SAXS profile (Figs. 2D and 3A–E).

The improvement of the results obtained as the number of conformations increases is reflected in the fit score values (*χ*^*2*^) obtained with a single-state model (2.29 ± 5.07), a two-state model (1.77 ± 4.77), or a four-state model (1.73 ± 0.11). The five-state model did not improve the *χ*^*2*^ fit over the four state model but decreased the error to ± 0.01. The representative conformations with higher contribution to the calculation display a large difference in the relative position of the CTD, evidenced by the value of the R_g_ ranging from 19 to 28 Å (Table S2). This result corroborates the hypothesis that the CTD possesses a high flexibility in solution and that an ensemble of conformations is required to best fit the solution data. The 5 Nsp1 models obtained with MultiFoXS are shown, in the same order as their corresponding R_g_ values (Fig. 3A–E, Table S2).

To complement this analysis, a homology model was obtained using AlphaFold2 (Bertoline et al. 2023) (Fig. S2A). This model was superimposed on the NTD with the closest model obtained from MultiFoXS (model #4, rmsd = 4.09 Å) (Fig. S2B and C). Regardless of the specific position of the CTD in each model, the relative position is quite similar. Furthermore, both models give very similar Rg (17.75 for AlphaFold2 model and 19.05 for MultiFoXS model), while the fitting of the AlphaFold2 model alone over the SAXS data were not good (*χ*^*2*^ = 12.84).

### Nsp1 dynamics

The structural flexibility observed in the CTD of Nsp1^SARS−CoV2^ ranging from residues 128 to 180 hinders the elucidation of the crystallographic structure of the full-length protein. The structure of the NTD was determined by X-ray crystallography (Semper et al. 2021; Clark et al. 2021) and NMR (Almeida et al. 2007), while the structure for the CTD was obtained only by cryo-EM in complex with the 40S ribosomal subunit (Shi et al. 2020; Thoms et al. 2020; Yuan et al. 2020).

Recently, the structure for the full length Nsp1, solved by NMR was published (Wang et al. 2023). In all 10 conformations reported in the study, the CTD (residues 128 to 180) was interacting with the NTD, presenting no secondary structure at all. Furthermore, when these structures were used alongside our SAXS data for an analysis performed with MultiFoXS, the conformations did not fit well with the experimental data (*χ*^*2*^ = 24.79), giving a calculated R_g_ value of 15.84 Å.

To study the different conformations that Nsp1^SARS−CoV2^ can explore, 4 replicas of 120 ns Molecular Dynamics (MD) simulations were performed for the full-length protein. We used MODELLER to build an initial model of the full-length Nsp1^SARS−CoV2^ protein (Fig. 3F) with a fully elongated loop (NC-loop) linking the NTD and CTD. As templates, we used the crystallographic structure of the NTD (residues 11 to 125) (Clark et al. 2021), and the coordinates for the CTD (residues 149 to 178) from the cryo-EM structure for the complex with the human 40S ribosomal subunit (Shi et al. 2020). This allowed us to obtain an initial conformation with a more elongated NC-loop than model 5 obtained with MultiFoXS (Fig. 3F).

**Fig. 4:**
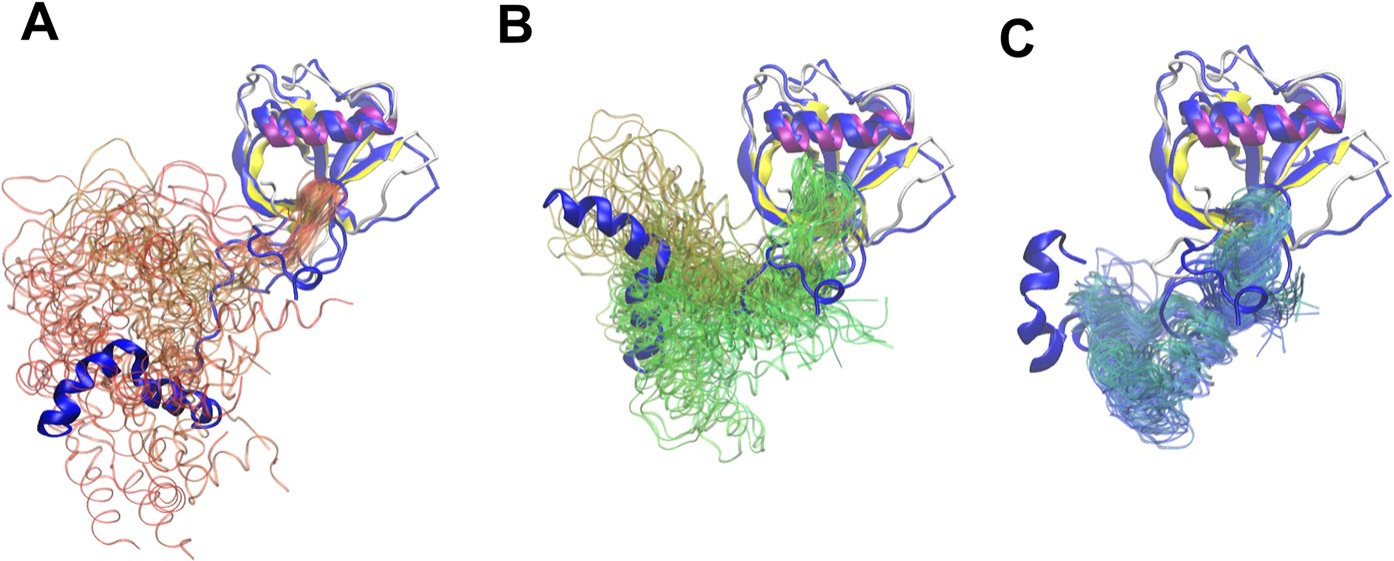
Time lapse representation for trajectories obtained from replica 4. For each panel, 25 equally spaced frames ranging over the specified time range are depicted. Residues 1 to 128 are displaced as thick ribbons and colored by secondary structure (purple for α-helix and yellow for β-strands), and residues 129 to 180 are displaced as thin ribbons and RGB colored from the beginning to the end of simulation time, respectively. In each panel, the closest conformation from the models obtained with MultiFoXS (thick blue ribbons) was superimposed, choosing for the alignment the backbone ⍺-carbon atoms ranging residues 15 to 120. **A** Alignment for model 5 and snapshots from the first 20 ns of replica 4. **B** Alignment for model 3 and snapshots from 20 to 60 ns simulation time. **C** Alignment for model 4 and snapshots from 60 to 120 ns simulation time. (Color figure online)

As the SAXS results indicate, the average shape of Nsp1^SARS−CoV2^ is elongated globular-like, with an estimated molecular weight of 24 kDa, and a radius of gyration of 22.64 Å and a maximum diameter of 78 Å. The initial conformation with a fully elongated NC-loop slightly exceeds 100 Å, while the diameter of the Nsp1 NTD measured from the crystal structure is ~ 40 Å, and the diameter for the most globular conformation obtained with MultiFoXS is ~ 55 Å. These data suggest that in solution the average structure of Nsp1^SARS−CoV2^ will neither display closely interacting NTD and CTD nor be elongated with a fully extended NC-loop, but rather assume an ensemble of structures where the protein is able to explore a broad conformational diversity (Fig. 4).

In classical MD simulations, the system always evolves towards a conformation that optimizes all interactions (i.e., converging towards an energy minimum). Notwithstanding, by studying the dynamics of 4 replicas of the Nsp1^SARS−CoV2^ full-length model, starting from the situation where the Nsp1^SARS−CoV2^ NTD and CTD are more distant (Fig. S3), we were able to sample the intermediate protein conformations between the elongated and the globular conformations as the CTD approaches the NTD. Comparison of the trajectories of our replicas with the models obtained with MultiFoXS, revealed that Nsp1^SARS−CoV2^ was able to visit conformations resembling all the 5 models used as a reference (Figs. 4, S3, S4).

To further assess whether our MD simulations agree with the experimental data, the R_g_ for each replica as a function of simulation time was calculated (Fig. S3). Through the analysis of R_g_ values it is possible to describe 3 different stages: for the first 20 ns, the R_g_ rapidly diminishes, corresponding to the NTD and CTD getting closer (Fig. 4). Next, the behavior in the range of 20–50 ns is heterogeneous among the 4 replicas, as can be evidenced by their SD value (Fig. S3). After 60 ns simulation time, the tertiary structure appears to be converging towards an ensemble of conformations that presents an R_g_ value in the range represented by the first 4 models obtained with MultiFoXS (Fig. 3A–D, Table S3).

With the hypothesis that the NTD and CTD of Nsp1^SARS−CoV2^ occasionally interact with each other, protein–protein docking studies were performed to evaluate a possible preference for binding interfaces. For the docking of the CTD over the NTD two approaches were adopted: the polypeptide chains were either treated as rigid bodies (Yan et al. 2020) or allowed to explore different conformations (flexible docking; HPEPDOCK (Zhou et al. 2018)). In both cases, a clear preference for two specific regions of the NTD was evidenced. To further understand the physicochemical aspects governing the interactions between the two Nsp1^SARS−CoV2^ domains and how they might occasionally interact, an electrostatic potential calculation was performed using the Adaptive Poisson-Boltzmann Solver (APBS) (Jurrus et al. 2018). To illustrate the results, the electrostatic potential is mapped over the protein surface for each domain separately (Fig. S5). The results show that the CTD is mostly negatively charged, while the NTD presents a positively charged region spanning the surface where CTD preferentially binds in the protein–protein docking results (Fig. S5A–E).

The N- and CTD of Nsp1^SARS−CoV2^ present different structural dynamics and diverse physicochemical characteristics. The last residue on the C-terminal end of the crystallographic structure of Nsp1^SARS−CoV2^ (residue 127) is located near the positively charged region on the NTD. Consequently, it is expected that the CTD would be in close proximity to this region. However, in the protein–protein docking studies presented here, no structural link between the NTD and CTD is observed, and thus no bias is introduced.

### SARS-CoV2 Nsp1 nuclease activity

**Fig. 5:**
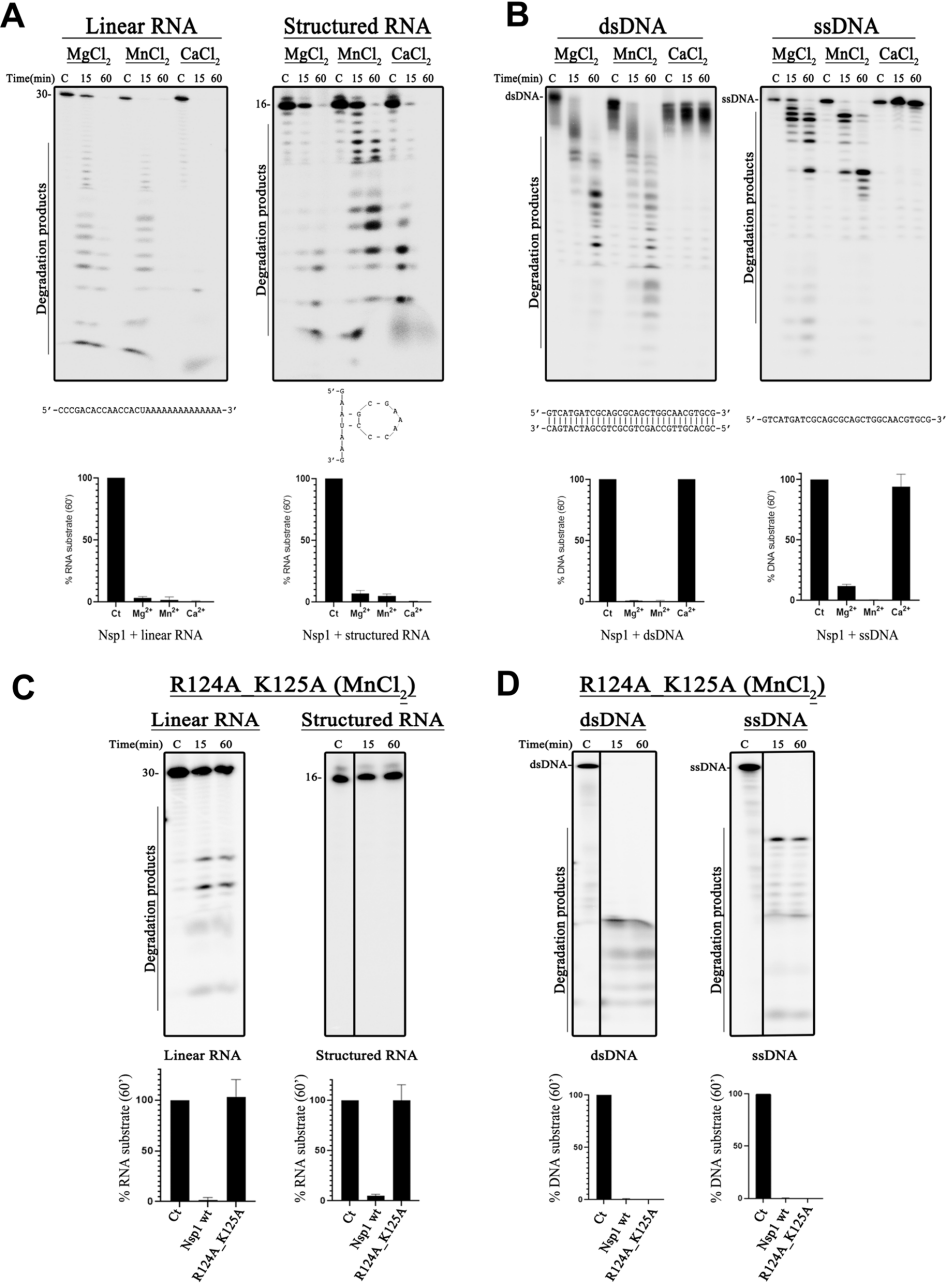
Nsp1 ^SARS−CoV2^ nuclease activity. **A** Nuclease activity using 50 nM of 30-mer (left panel) and 16-mer (right panel) RNA substrates. **B** Nuclease activity using 50 nM of dsDNA (left panel) and ssDNA (right panel) substrates. Nsp1 ^SARS−CoV2^ was used at 5 µM concentration in all panels. **C** Nuclease activity of Nsp1 ^SARS_CoV2^ R124A_K125A variant version using 50 nM of 30-mer RNA (left panel) and 16-mer RNA (right panel) substrates. **D** Nuclease activity of the double variant R124A/K125A Nsp1 ^SARS−CoV2^ using 50 nM of ssDNA (left panel) and dsDNA (right panel) RNA substrates. The double variant R124A/K125A Nsp1 ^SARS−CoV2^ was used at 5 µM concentration in all assays. For all panels, reactions were analyzed on 7 M urea/20% polyacrylamide gels. “*C”*, denotes control reactions in the absence of Nsp1 ^SARS−CoV2^ or the double variant R124A/K125A Nsp1 ^SARS−CoV2^ for the time point of 60 min; time points are indicated in the top of each panel. The metal concentration used was 10 mM. Disappearance of substrate (RNA or DNA) along time was quantified using the ImageQuant (Cytiva). GraphPad Prism 8 software. The sequence of each substrate is indicated at the bottom of each panel. The structure of each RNA molecule was predicted using Mfold RNA modelling server (http://www.unafold.org/RNA_form.php)

It is well-established that Nsp1 physically interacts with ribosomes to stop host translation either by blocking the 40S subunit of the ribosome to end the host protein translation or through endonucleolytic cleavage near the 5′ UTR of the host mRNA, thus making them incompetent for translation (Huang et al. 2011; Lokugamage et al. 2012; Abaeva et al. 2023; Tardivat et al. 2023; Shehata and Parker 2023). However, it remains crucial to elucidate the mechanism underlying of RNA degradation induced by Nsp1, particularly whether this protein is the responsible for the previously described endonucleolytic activity, which was not yet fully demonstrated (Abaeva et al. 2023; Tardivat et al. 2023; Shehata and Parker 2023). As such, we examined the in vitro nuclease activity of Nsp1^SARS−CoV2^ using two different RNA oligonucleotides as substrates: a 30-nucleotide long and linear RNA molecule (30-mer), and a single-stranded RNA with a stem-loop (16-mer) (Fig. 5A). The results showed that Nsp1^SARS−CoV2^ is able to efficiently cleave both RNA substrates at multiple sites in the presence of Mg^2+^, Mn^2+^ and Ca^2+^(Fig. 5A). Strikingly, the enzyme seems to be more active in the presence of Ca^2+^, as the substrate consumption was faster.

In order to evaluate the RNA degradation efficiency of Nsp1^SARS−CoV2^, an assay was performed using protein concentrations from 0.05 μM to 5 μM. Our results indicate that at 0.2 μM the protein already presents residual activity, and at 0.5 μM it is sufficient to degrade the RNA molecule (Fig. S6).

We also tested a wide range of Mg^2+^, Mn^2+^ and Ca^2+^ concentrations (0.5 to 10 mM) using the 16-mer RNA substrate to determine the optimal conditions for Nsp1^SARS−CoV2^ activity (Fig. S7A and B). The results demonstrated that Nsp1 is active under all the conditions tested, with an increase in cleavage efficiency proportional to the metal cofactor concentration, reaching a maximum when 10 mM of each ion was present in the reaction (Fig. S7A and B).

Significantly reduced enzyme activity was observed when Nsp1^SARS−CoV2^ was tested for 16-mer RNA degradation in the absence of any metallic cofactor (Fig. S7A and B).

Moreover, we tested whether Nsp1^SARS−CoV2^ can cleave DNA molecules, using two different DNA substrates: a double-stranded (dsDNA) and a single-stranded DNA (ssDNA) oligonucleotide (Fig. 5B). Nsp1^SARS−CoV2^ was shown to non-specifically cleave DNA molecules (dsDNA or ssDNA) at multiple sites, exhibiting activity only in the presence of Mg^2+^ and Mn^2+^, being Mn^2+^ the preferred cofactor. A different behaviour is seen in the presence of Ca^2+^, where Nsp1^SARS−CoV2^ is unable to degrade any DNA substrate, remaining unaltered after 60 min of incubation with the enzyme, similar to the control reaction (Fig. 5B). The concentrations of Ca^2+^ used in the assay ranged from 0.5 to 10 mM (Fig. S7C and D) and no activity could be observed. This confirmed that Nsp1^SARS−CoV2^ does not promote DNA degradation in the presence of Ca^2+^. In contrast, with both Mg^2+^ and Mn^2+^, the results showed that Nsp1^SARS−CoV2^ is active at all the metal concentrations tested (ranging from 0.5 to 10 mM), with 10 mM of either metal appearing to be the concentration at which the activity is higher under the conditions tested (Fig. S7C and D). As a control, a reaction in the absence of any metal was also performed, and in contrast to what was observed for the RNA substrates, Nsp1^SARS−CoV2^ was unable to cleave dsDNA under these conditions (Fig. S7A–C). Therefore, the nuclease activity of Nsp1^SARS−CoV2^ over DNA substrates seems to be firmly dependent on the presence of metal ions, such as Mg^2+^ or Mn^2+^.

## Inactive variant of Nsp1

It has been previously suggested that R124 and K125 (located in the NTD region) could be involved in Nsp1^SARS−CoV2^ activity (Lokugamage et al. 2012; Bujanic et al. 2022; Abaeva et al. 2023; Tardivat et al. 2023; Shehata and Parker 2023). Thus, to understand the molecular mechanism associated with the nuclease activity towards RNA and DNA substrates, the double Nsp1^SARS−CoV2^ variant R124A/K125A was generated. The oligomeric state of the Nsp1 R124A/K125A protein was analysed by SEC, revealing a lower elution volume compared to the wild-type protein, corresponding to 1.6 ± 0.2 subunit equivalents suggesting that the protein may still retain its monomeric form but in a different conformation as the native protein and with a higher hydrodynamic radius (Fig. S8A). Interestingly, the addition of manganese did had only a slight effect in this variant. The stability of the R124A/K125A variant protein was also assessed by thermal shift assays, and the results (Fig. S8B) show that this protein presents a T_M_ similar to that of the native protein, and unlike what was observed for the native protein, the different metals did not have a clear effect on the variant stability. However it is important to notice that the slope of the melting curves for the variant protein in the different conditions are lower than for the native protein (Fig. S8B).

The in vitro activity of this variant was then analysed using the same substrates and assay conditions as those employed for the native protein (Fig. 5C and D). Manganese was used as a co-factor, since it demonstrated increased activity for both substrates compared to magnesium, being the preferred co-factor in all assays with Nsp1^SARS−CoV2^. The results demonstrated that the variant Nsp1^SARS−CoV2^ R124A/K125A lost its ability to efficiently cleave both RNA molecules tested, showing only residual activity against 30-mer linear RNA, and no activity for the 16-mer structured RNA. Surprisingly, the variant appears to be more prone to degrade DNA than the native protein, completely cleaving both ssDNA and dsDNA molecules after 60 min of incubation. The residues R124 and K125 were identified as important for RNA cleavage but not required for DNA degradation, suggesting different modes of action or different binding sites on Nsp1^SARS−CoV2^ for RNA and DNA.

## Discussion

In recent years, the world has faced an unprecedented situation due to the SARS-CoV2 coronavirus pandemic. Nsp1 is translated from the ORF1a/b polyprotein and is one of the first proteins expressed upon virus infection and has been proposed to be one of its virulence factors (HASÖKSÜZ et al. 2020; Gordon et al. 2020a, b; Bujanic et al. 2022). It is implicated in several cellular processes, such as the inhibition of translation, by promoting the host mRNA cleavage and hindering its nuclear export (Zhang et al. 2021; Simeoni et al. 2021; Abaeva et al. 2023; Tardivat et al. 2023; Shehata and Parker 2023). However, the molecular mechanisms underlying these actions are not yet fully understood.

To further deepen our knowledge on Nsp1^SARS−CoV2^ biological function, we have addressed its biochemical and biophysical properties in presence of different metals, including calcium, magnesium, and manganese. These metals are known to play fundamental roles during viral infection (Chaturvedi and Shrivastava 2005; Li et al. 2022).

The effect of these metals on the overall structure of Nsp1^SARS−CoV2^ was evaluated. Structurally, this protein is composed by a NTD that connects to a flexible CTD by a loop (Züst et al. 2007; Yuan et al. 2020; Kumar et al. 2021). Our CD and SAXS data support this flexibility, and suggests an elongated shape in the native state, and when incubated with Ca^2+^ or Mg^2+^ (Figs. 1, 2, 3, and 4). Notably, in the presence of Mn^2+^, Nsp1^SARS−CoV2^ is proposed to adopt a globular conformation, potentially involving an interaction between the NTD and CTD (Fig. 1). It is quite interesting that the NTD presents an overall positive charge while the CTD has a negative charge, indicating that these domains may interact with each other under various conditions or with different substrates (Fig. S5). This suggests that Mn^2+^ is an important player, as it is a metal capable of mediating the contact between the Nsp1^SARS−CoV2^ domains, causing the protein to acquire a globular conformation (Fig. 1).

The analysis of potential metal binding sites for various metals using IonCom server (Hu et al. 2016), suggests that the residues Asp33, Glu37, and Glu41 which are proximal located *ca.* 5 Å to each other, may form a metal binding site. Notably, Asp25 is located approximately 23 Å away from both Asp33 and Glu37, indicating that Asp25, together with other nearby residues like Gln22 and Glu113, could potentially constitute a different metal binding site (Fig.S9). However, it is important to note that further studies are needed to fully elucidate and characterize these different metal binding sites.

Previous studies have shown that Nsp1 inhibits the protein translation process (Lokugamage et al. 2012; Thoms et al. 1979; Schubert et al. 2020), and that the Nsp1^SARS−CoV2^ CTD binds to the mRNA entry channel of the 40S ribosome subunit, physically blocking it (Yuan et al. 2020, 2021). This interaction with the ribosome induces endonucleolytic cleavage of cellular mRNAs, thereby accelerating their turnover (Abaeva et al. 2023; Tardivat et al. 2023; Shehata and Parker 2023). Furthermore, it has been recently demonstrated that Nsp1^SARS−CoV2^ induces mRNA cleavage in the 5´UTR regions (Tardivat et al. 2023). This degradation was proposed to require an interaction between the mRNA and the ribosome (Abaeva et al. 2023; Shehata and Parker 2023). However, it was unknown if Nsp1^SARS−CoV2^ could have endonucleolytic activity in the absence of the ribosome.

We have unveiled, for the first time, the nucleolytic activity of Nsp1^SARS−CoV2^ for both RNA and DNA, in a metal-dependent manner and in the absence of ribosome, establishing its role in endonucleolytic cleavage of the host mRNAs. The protein degrades RNA molecules in the presence of Mg^2+^, Mn^2+^ or Ca^2+^ (Figs. 5, S7), appearing to be more active with Ca^2+^, similarly to what was previously demonstrated for a few other ribonucleases (Grünberg et al. 2021). Furthermore, DNA cleavage occurs in the presence of Mg^2+^ or Mn^2+^, with no degradation in the presence of Ca^2+^ (Figs. 5, S7).

These results suggest that the in vivo activity of Nsp1^SARS−CoV2^ may be modulated by the presence of different metals, namely calcium, manganese, and magnesium. The alterations in the catalytic activity with the different metal ions could potentially act as a functional switch in vivo. Such a mechanism might play a crucial role during viral infection, as different metal ions released from the different compartments of the cell during different phases of the viral infection could contribute to changes in RNA *versus* DNA cleavage (Chaturvedi and Shrivastava 2005; Li et al. 2022).

The double variant R124A/K125A Nsp1^SARS−CoV2^ is unable to degrade RNA, while DNA cleavage remains unaffected. This supports the notion that the residues R124 and K125 are located in a positively charged region in the electrostatic surface of the Nsp1 structural model (Figs. 5 and S5F–H). The substitution of these residues to alanines reduces the positive surface charge, and thus may explain the decrease of the RNase activity observed for this variant (Fig. S5). Previous studies have shown that the endoribonucleolytic activity is blocked for the R124 or K125 single variant of Nsp1^SARS−CoV^ (Lokugamage et al. 2012) and that the double variant of Nsp1^SARS−CoV2^ also lacks the ability to degrade RNA (Mendez et al. 2021; Abaeva et al. 2023; Shehata and Parker 2023). Moreover, these residues are proposed to be involved in strong ionic interactions with RNA, and the mutations to alanines promotes the decrease of interaction with RNA (Vankadari et al. 2020). Additionally, in a study proposing potential drug-binding pockets, these residues are located in a pocket suggested to be involved as RNA binding site (Borsatto et al. 2022). We propose that the DNA binding site could correspond to one of the other pockets described by the authors (Borsatto et al. 2022), providing a plausible explanation for the retained DNA nucleolytic activity of the R124A/K125A variant. It is also noteworthy that Arg and Lys residues are commonly associated with nuclease activity in several other nucleases [e.g. for review (Yang 2011)]. Interestingly these two residues are mostly conserved in all the Nsp1 from Coronaviruses (Min et al. 2020).

It has been previously described that during viral infections there are changes and fluctuations in the concentrations of different metal ions in the host cells (da Silva and Williams 2001; Chaturvedi and Shrivastava 2005; Zhou et al. 2009; Zevini et al. 2017; Chen 2018; Wang et al. 2018; Chang-Graham et al. 2019; Iotti et al. 2020; Li et al. 2022; Berlansky et al. 2022). However, a direct link between Nsp1^SARS−CoV2^ and metal homeostasis has not been reported. Our data supports a metal dependent mechanism for Nsp1^SARS−CoV2^ linking metal ions to the nucleolytic activity.

The role of calcium in the host during coronavirus infection has been previously described (Berlansky et al. 2022), notably an increase in intracellular calcium concentration occurs in the cytosol. We suggest that Nsp1^SARS−CoV2^ primarily functions as a ribonuclease, with its activity modulated by calcium in this cellular compartment. According to our results, under calcium increase, Nsp1^SARS−CoV2^ is expected to completely degrade both linear and structured RNA. During viral infection, manganese also plays a significant role, as it is exported from the mitochondria to the cytosol to activate the INL αβ pathways, resulting in a *ca.* 60-fold increase in its concentration (Wang et al. 2018). Interestingly, and according to our data, in the presence of manganese, Nsp1^SARS−CoV2^ endonuclease presents different specificity in cleaving linear and structured RNAs. However, the biological significance of this observed difference needs to be further clarified, especially its connection with the mechanism by which Nsp1 degrades host mRNA rather than the viral mRNA.

The DNase activity of Nsp1^SARS−CoV2^ reported in this work adds further evidence to a possible interaction with the host cell replication machinery during viral infection. An interactome study between host and SARS-CoV2 proteins suggested a potential association between Nsp1 and the primosome, responsible for initiating DNA synthesis (Chen et al. 2021). The primosome was identified as an interactor for Nsp1^SARS−CoV^ (Gordon et al. 2020a). Furthermore, a cryoEM study demonstrated a structural interaction between Nsp1^SARS−CoV^ and the catalytic subunit of the primosome involving the N-terminal domain (Kilkenny et al. 2022). In addition, we have reported that Nsp1^SARS−CoV2^ is only able to cleave DNA molecules in the presence of Mn^2+^ or Mg^2+^ ions; manganese has been proposed to be present in the nucleus and magnesium is known to stabilize DNA and chromatin structures and is known as a cofactor of enzymes involved in DNA processing (Naora et al. 1961; Romani and Scarpa 1992; Hartwig 2001; Chen 2018). Therefore, this could represent an additional layer of regulation through Nsp1^SARS−CoV2^.

The various studies so far reported, suggested different functions of Nsp1, rendering it as a multifunctional enzyme. However, the key question that remains to be addressed is how all these possible functions are regulated. The data presented in this study reveal, for the first time, that Nsp1^SARS−CoV2^ alone is able to directly degrade both RNA and DNA molecules and we have characterized its endonucleolytic activity. We showed that this activity is intricately modulated by the presence of different metals, which are triggered in response to viral infection. In summary, this study establishes a connection between fluctuations/presence in metal ion concentrations within host cell and specific functional dynamics of Nsp1^SARS−CoV2^ during viral infection. However, whether fluctuations in the metal concentration also regulate the distribution of the enzyme in different cellular compartments remains to be elucidated.

## Material and methods

### Protein expression and purification

The gene coding for Nsp1^SARS−CoV2^ cloned into pET28a( +)TEV plasmid using codons optimized for expression in *Escherichia coli*, was obtained from Genscript, Netherlands.

The mutant of *nsp1* was obtained using a standard site-directed mutagenesis protocol, using the NZYtech mutagenesis kit according to the manufacturers protocol and the primers FPR124AK125R: 5´GTGCTGCTGGCGGCGAACGGTAACAAGGGT-3´ and RPR124AK125R: 5´ACCCTTGTTACCGTTCGCCGCCAGCAGCAC-3. The mutations were confirmed by sequencing (Eurofins genomic).

The *E. coli* BL21(DE3) transformed with pET28a( +)TEV-Nsp1 and double variant were expressed in Luria Broth (LB) medium containing 50 μg mL^−1^ kanamycin at 37 °C, inducing with 100 μM of Isopropyl β-d-1-thiogalactopyranoside (IPTG) at an optical density at 600 nm of 0.6. Cells were harvested by centrifugation and resuspended in 20 mM HEPES pH 7.0, 10% glycerol, 300 mM NaCl, 50 mM L-Arginine, 50 mM L-glutamic acid, 10 mM MgCl_2_, 1 µg mL^−1^ DNase I and 0.1 mg mL^−1^ lysozyme and disrupted by 3 cycles in a French press cell at 1000 psi.

The crude extracts were subjected to a low-speed centrifugation at 15,000 rpm for 45 min at 4 °C. The supernatant was loaded onto a His Trap EXCEL column (Cytiva), using the binding buffer 20 mM HEPES pH 7.0, 10% glycerol, 300 mM NaCl, 50 m M L-Arginine, 50 mM L-glutamic acid and 10 mM imidazole. The protein was eluted with the binding buffer supplemented with 1 M imidazole. The sample containing Nsp1 was loaded onto a desalting column (Hi prep 26/10 Desalting (Ge Healthcare) equilibrated with the buffer 20 mM HEPES pH 7.0, 10% glycerol, 300 mM NaCl, 50 mM L-Arginine, 50 mM L-glutamic acid. The His-Tag was cleaved by adding TEV protease during an overnight incubation at 4 °C, followed by loading onto a HisTrap EXCEL column using the same buffers as mentioned above. The protein was eluted in the flow-through. Nsp1 was further purified by size exclusion chromatography (Superdex 75 10/300 GL, Cytiva), with the buffer 20 mM HEPES pH 7.0, 10% glycerol, 300 mM NaCl, 50 mM L-Arginine, 50 mM L-glutamic acid. Protein purity was analysed by SDS-PAGE. The protein was concentrated using the 10 kDa cut-off Amicons (Merck Millipore). The N-terminal sequence of the proteins were confirmed by Edman degradation using an Applied Biosystem model PROCISE 494 protein sequencer (Edman and Begg 1967). The addition of equimolar amounts of glutamate and arginine has been suggested to increase solubility and stability buffers (Golovanov et al. 2004), and this approach was previously tested for Nsp1^SARS−CoV2^ buffers (Clark et al. 2021). Consequently, these two additives were included in the lysis and purification buffers.

### Thermal shift assay

Fluorescence based thermal shift assay was carried out on an iCycle iQ5 Real Time PCR Detection System (Bio-Rad), equipped with a charge-coupled device (CCD) camera and a Cy3 filter with excitation and emission wavelengths of 490 and 575 nm, respectively. The reaction mixture (22 µL) containing 7.5 µg of Nsp1 purified protein, 25 × Sypro orange dye (Molecular Probes) and different buffers was subjected to thermal denaturation on a 96-well plate (low profile plate, Bio-Rad), that were sealed with Optical Quality Sealing Tape (Bio-Rad) and centrifuged at 2500 g for 2 min immediately before the assay to remove possible air bubbles. The thermal denaturation was performed from 20 °C to 90 °C with an increment of 0.5 °C min^−1^. The 96-buffer formulation screen used was home-made and the Hampton Research additive screenHTt96 was used for the additive complement for protein stabilization. The screening conditions included various divalent metals, such as Mg, Ca, Mn, Fe, Co, Ni, Cu, Zn. However, with the exception of Mg, Ca, and Mn, protein precipitation occurred in all the other conditions. Consequently, subsequent assays were exclusively performed in the presence of Mg, Ca, and Mn.

Data were fitted to a standard four parameter Boltzmann sigmoidal equation.

### Circular dichroism

CD (Circular Dichroism) spectra were measured using a JASCO J-850 CD spectrometer connected to a Peltier temperature controller. CD measurements were carried out in a cuvette with a 0.1 cm path length. The scanning speed was 100 nm min^−1^ with a bandwidth of 1.0 nm and a response time of 0.5 s. The spectra were obtained from 198 to 260 nm and the experiments were repeated two times. All the assays were performed in 20 mM sodium phosphate buffer pH 6.5, with a protein concentration of 0.1 mg mL^−1^. The protein samples were incubated with stoichiometric concentration of the different metals (Nsp1-Mg 1:1; Nsp1-Mn 1:2 and Nsp1-Ca 1:4). The percentage of the different secondary structure folds were calculated using single spectra analysis in Beta Structure Selection–BeStSel–webserver (Micsonai et al. 2022a, b).

### Size exclusion chromatography analyses

The oligomerization state was determined using a Superdex 200 column (10/300 GL, GE Healthcare) using different buffers at room temperature. The column was previously calibrated using standard proteins ranging from 14 to 660 kDa (GE Healthcare). The different samples analysed were incubated for 10 min prior to chromatography.

### Isothermal calorimetry

ITC experiments were performed using an ITC200 Microcalorimeter from MicroCal, LLC. The concentration of Nsp1 protein was 100 µM, and that for metal was 1 mM for Ca^2+^, 2 mM for Mg^2+^ and 0.7 mM for Mn^2+^ using the buffer 50 mM HEPES pH7.0 and 250 mM NaCl. In each individual experiment, ~ 38 µL of metal was injected through the computer-controlled 40-µL micro-syringe (cell volume = 250 µL) while stirring at 600 rpm at 25 °C. The heat generated in each injection of the metal solution aliquot decreased with each additional injection, producing a typical titration isotherm. As a control, the ITC experiments were performed identically with only buffer-protein and buffer-metal. The experimental data were fitted to a theoretical titration curve using the software OriginLab standard one-site model was used with *ΔH* (enthalpy change, in kcal mol^−1^), *ΔS* (entropy change, in cal mol^−1^.K). *K*_*D*_ (dissociation constant, in nM), *n* (number of binding sites) as the variables. The interaction parameters such as dissociation constant (*K*_*D*_), enthalpy change (*ΔH*), entropy change (*ΔS*) and number. A of metal binding sites (*n*) are summarized in Table 1.

### SAXS data collection, processing, and ab initio modeling

SAXS data were collected at the ESRF BioSAXS beamline BM29 (Tully et al. 2023). An inline HPLC system (Shimadzu) was used (Tully et al. 2021) coupled directly to the BM29 via a vacuum capillary. The protein concentration used was 7.5 mg mL^−1^ in the buffer 20 mM HEPES pH 7.0, 250 mM NaCl, 10% (w/v) glycerol, 50 mM L-arginine and 50 mM glutamic acid. A Superdex 200 10/300 GL (GE Healthcare) was equilibrated with buffer at room temperature. SAXS data were collected using X-rays of wavelength of 0.9919 Å (12.5 keV) and a sample-to-detector distance of 2.81 m corresponding to a q-range of 0.007–0.5 Å^−1^, where q is the magnitude of the scattering vector given by q = 4π/λ sin (θ), with 2θ the scattering angle. A total of 1200 frames (at 2 s/frame) were collected for the protein sample. A second protein sample was tested in a buffer containing 50 mM MnCl_2_. However, during X-rays exposure, the critical dose for radiation damage was exceeded that led to severe precipitation being observed in the capillary (Hopkins and Thorne 2016). This was initiated by increased ionisation from the high concentration of metal ions in the mobile phase when exposed with X-rays rather than from the protein itself. This was particularly evident in the presence of manganese. Consequently, no SAXS could be obtained for the protein incubated with manganese.

The beamline integrated automated processing pipeline, using FreeSAS, integrated each individual frame (Kieffer et al. 2022) before buffer subtraction, and primary data processing was carried out manually using Scatter IV (Tully et al. 2021). The SEC-SAXS was unable to completely separate the protein from the aggregates, leading to overlapping peaks. To determine the number of distinct scatterers and extract the individual components, the program BioXTAS RAW, which encompasses a single value decomposition (SVD) with evolving factor analysis (EFA) tool was used (Meisburger et al. 2016; Tully et al. 2021).

### Nsp1 models

To obtain a set of models in the best possible agreement with the SAXS results we used MultiFoXS (Schneidman-Duhovny et al. 2016). This utility allows the use of multiple structures representative of the wide conformational space that the protein is able to sample, giving the best fitting to the experimental SAXS profile provided. As input, we used the crystallographic structure of the NTD of Nsp1^SARS−CoV2^ at 1.77 Å resolution (residues 10 to 127, PDB ID 7K7P) (Clark et al. 2021), the SAXS profile data obtained from our measurements with recombinant Nsp1, and defining the first 9 residues in the NTD as flexible, and the region comprising the CTD along with the loop linking both domains (residues 128 to 180).

### Molecular dynamics simulations

MODELLER software (Webb and Sali 2017) was used to obtain the initial 3D model of the full-length Nsp1^SARS−CoV2^ (ID PRO_0000449619), using as templates the crystallographic structure for the NTD (residues 11 to 125; PDB ID 7K7P; (Clark et al. 2021), and the coordinates for the C-terminal domain (residues 149 to 178) from the cryo-EM structure of the complex with the human 40S ribosomal subunit (Shi et al. 2020); PDB ID 7K5I).

From the best model obtained, the system was prepared to perform MD simulations using the AMBER18 package (Salomon-Ferrer et al. 2013). The initial model was analyzed with ERRAT, giving a reasonable overall quality factor of 90.6. The AMBER14SB force field was chosen to assign parameters to all protein residues. The Nsp1 model was placed into a truncated octahedral box of TIP3P water molecules, with a 15 Å distance between the border of the box and the closest atom of the solute. The total system was composed of 180 protein residues and 46,476 water molecules, for a total of 142,196 atoms. All the systems were optimized with an energy minimization step consisting of 10,000 cycles using the steepest descent algorithm and 10,000 cycles with conjugate gradient minimization. The temperature was increased from 0 to 10 K in a 10 ps constant volume MD with a 0.1 fs time step, and a harmonic restraint potential of 10 kcal mol^−1^ Å^−2^ applied over all protein residues in the complex. Thereafter, the temperature was increased from 10 to 300 K in a 50 ps constant volume MD with a 0.5 fs time step, applying a force constant of 5 kcal mol^−1^ Å^−2^ to the protein backbone atoms. After the samples had been heated, the density was equilibrated with a 100 ps MD simulation at constant temperature and pressure with a time step of 1 ps and applying a force constant of 1 kcal mol^−1^ Å^−2^ to the protein backbone atoms, followed by a 10 ns equilibration in the same conditions, but applying a restraint with a force constant of 1 kcal mol^−1^ Å^−2^ over backbone atoms of residues 1 to 125 and 149 to 180. To control the temperature, a Langevin thermostat was used, whereas a Berendsen barostat was chosen to adjust the pressure to 1 bar (both regulated every 1 ps). For production MD, 100 ns replicas in the NTP ensemble were conducted, with a time step of 2 fs. All the simulations were performed under periodic boundary conditions (Essmann et al. 1995) using the SHAKE algorithm (Ryckaert et al. 1977) to keep hydrogen atoms at equilibrium bond lengths. Long-range electrostatic interactions were handled with Ewald sums, setting a cutoff distance of 10 Å. For the analysis of the trajectories (RMSD, distance analysis) cpptraj (Roe and Cheatham 2013) from AMBER18 was used. All molecular visualization and drawings were performed with the Visual Molecular Dynamics program (Humphrey et al. 1996).

### RNA and DNA 5’-end labelling

The following synthetic oligonucleotides (StabVida, Portugal) were used as substrates in the activity assays: 16-mer RNA (5’-GAAGCGAAACCCUAAG-3’); 30-mer RNA (5’-CCCGACACCAACCACUAAAAAAAAAAAAAA-3’); 31-mer DNA (5’-GTCATGATCGCAGCGCAGCTGGCAACGTGCG-3’). Each oligonucleotide was labelled at its 5′ end with [^32^P]-γ-ATP and T4 Polynucleotide Kinase (Ambion) in a standard reaction. MicroSpin G-50 columns (Cytiva) were used to remove the excess of [^32^P]-γ-ATP. In order to fold them into their secondary structures, the substrates were resuspended in 10 mM of Tris–HCl pH 8.0 and incubated 10 min at 80 °C followed by 30 min at 37 °C. The 31-mer DNA was also hybridized to the complementary non-labelled 31-mer oligonucleotide (5′-CGCACGTTGCCAGCTGCGCTGCGATCATGAC-3′) added in excess (molar ratio 1:10) in order to obtain a perfect 31-31ds duplex. The hybridization was performed during 10 min at 80 °C followed by 30 min at 37 °C. The formation of the DNA duplex was confirmed in 15% PAGE.

#### Activity assays

The in vitro activity assays were performed as previously described (Saramago et al. 2017) with a few modifications. The assays were in the presence of the activity buffer (20 mM HEPES pH 7.4 and 10 mM of either MgCl_2_, MnCl_2_ or CaCl_2_), and the Nsp1^SARS−CoV2^ protein (protein concentrations are indicated in the figure legends). The reactions were started by the addition of 50 nM of the substrate, and further incubated at 37 °C. Aliquots of 4 µl were withdrawn at the time-points indicated in the respective figures, and the reactions were stopped by the addition of a formamide-containing dye supplemented with 10 mM of EDTA. A control reaction containing only the RNA substrate and the activity buffer (without the enzyme) was incubated in the same conditions during the full time of the assay. Reaction products were resolved in a 20% denaturant polyacrylamide gel (7 M urea). Signals were visualized by PhosphorImaging (TLA-5100 Series, Fuji).

## Supplementary Information

Below is the link to the electronic supplementary material.

Supplementary file1 (PDF 1968 KB)

## Data Availability

The data underlying this article are available in the article and in its online supplementary material.
