# Supplementary material for: SARS-CoV2 Nsp1 is a metal-dependent DNA and RNA endonuclease
Source: Biometals. 2024 Mar 28;37(5):1127–46. doi: 10.1007/s10534-024-00596-z (PMC11473540; doi:10.1007/s10534-024-00596-z)
Supplement: Supplementary file 1 — Supplementary file1 (PDF 1968 KB) [file 10534_2024_596_MOESM1_ESM.pdf]

Supplementary Material

SARS-CoV2 Nsp1 is a metal-dependent DNA and RNA endonuclease

Bruno A. Salgueiro[1], Margarida Saramago[1], Mark D Tully[2], Federico Issoglio[1], Sara T. N. Silva[1], Ana C. F. Paiva[1,3], Cecília M. Arraiano[1], Pedro M. Matias[1,3], Rute G. Matos[1], Elin Moe[1,4*], Célia V. Romão[1,*]

1- ITQB-NOVA, Instituto de Tecnologia Química e Biológica António Xavier, Universidade Nova de Lisboa, Avenida da República, 2780-157 Oeiras, Portugal
2- ESRF, European Synchrotron Radiation Facility, 71, avenue des Martyrs CS 40220. 38043 Grenoble Cedex 9, France
3- iBET – Instituto de Biologia Experimental e Tecnológica, Apartado 12, 2780-901 Oeiras, Portugal
4- Department of Chemistry, UiT – the Arctic University of Norway, Tromsø, Norway

Figure S1

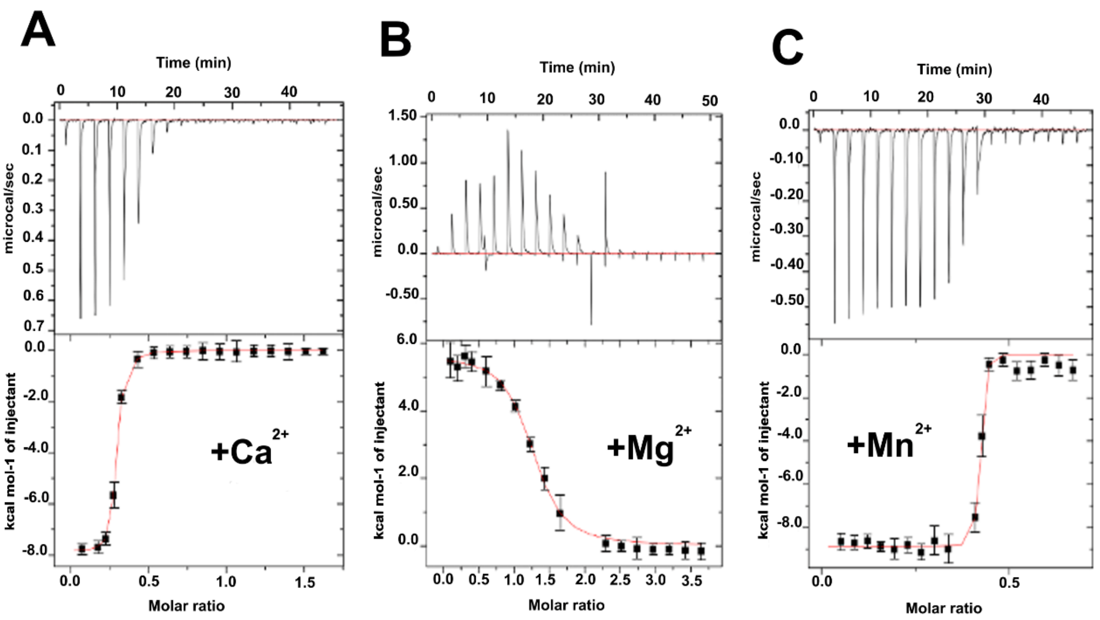

Figure S1. Isothermal titration calorimetry (ITC) of the Nsp1 $^{\text{SARS-CoV2}}$ protein with different metals. (A, B, C) ITC indicates direct binding of compound to $CaCl_2$, $MgCl_2$ or $MnCl_2$ to the Nsp1$^{\text{SARS-CoV2}}$, indicating a 4:1 **(A)**, 1:1 **(B)** and 2:1 **(C)** molar ratio, respectively.

Figure S2

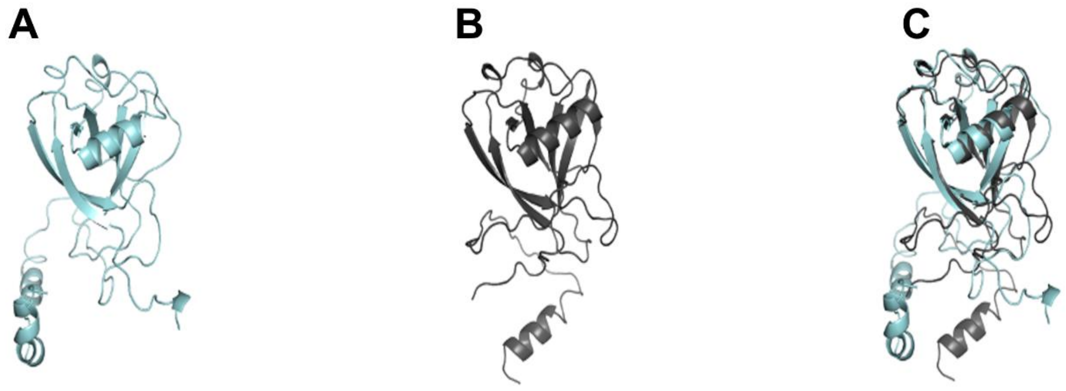

Figure S2. Structural model of Nsp1$^{SARS\text{-}CoV2}$ obtained with Alphafold2.ipynb and comparison with the model obtained with MultiFoXS. (A) Model #4 obtained with MultiFoXS. **(B)** Homology model obtained with AlphaFold2.ipynb **(C)** Superposition of model #4 obtained with MultiFoXS and Alphafold2.ipynb.

Figure S3

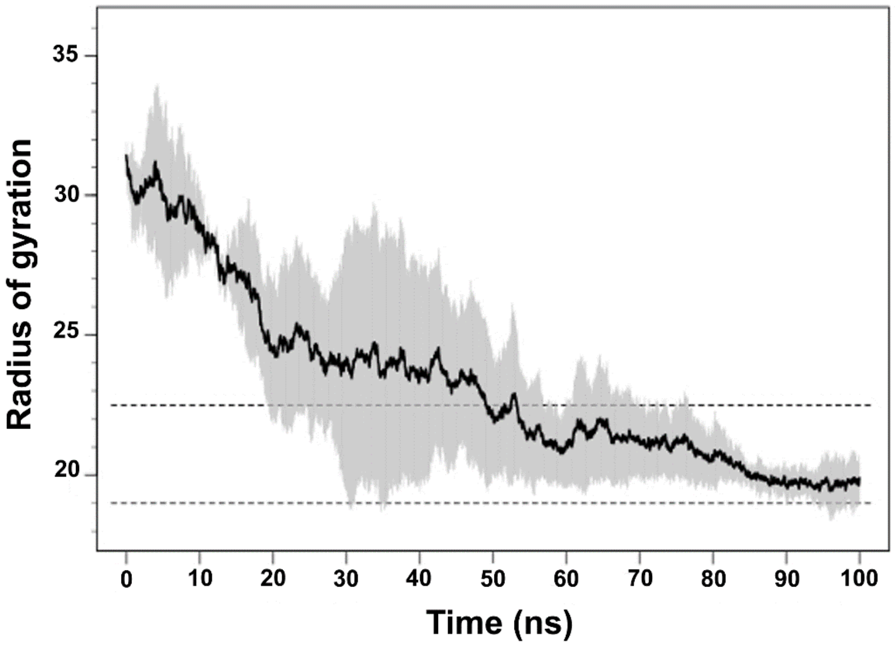

Figure S3. **Radius of gyration as a function of simulation time for the full length model of Nsp1 $^{SARS-CoV2}$**. In black lines the mean value and thin gray lines the standard deviation, obtained from 4 independent replicas. The dashed lines represent the range of R_g values for the models 1 to 4 obtained with MultiFoXS (see Table S2).

Figure S4

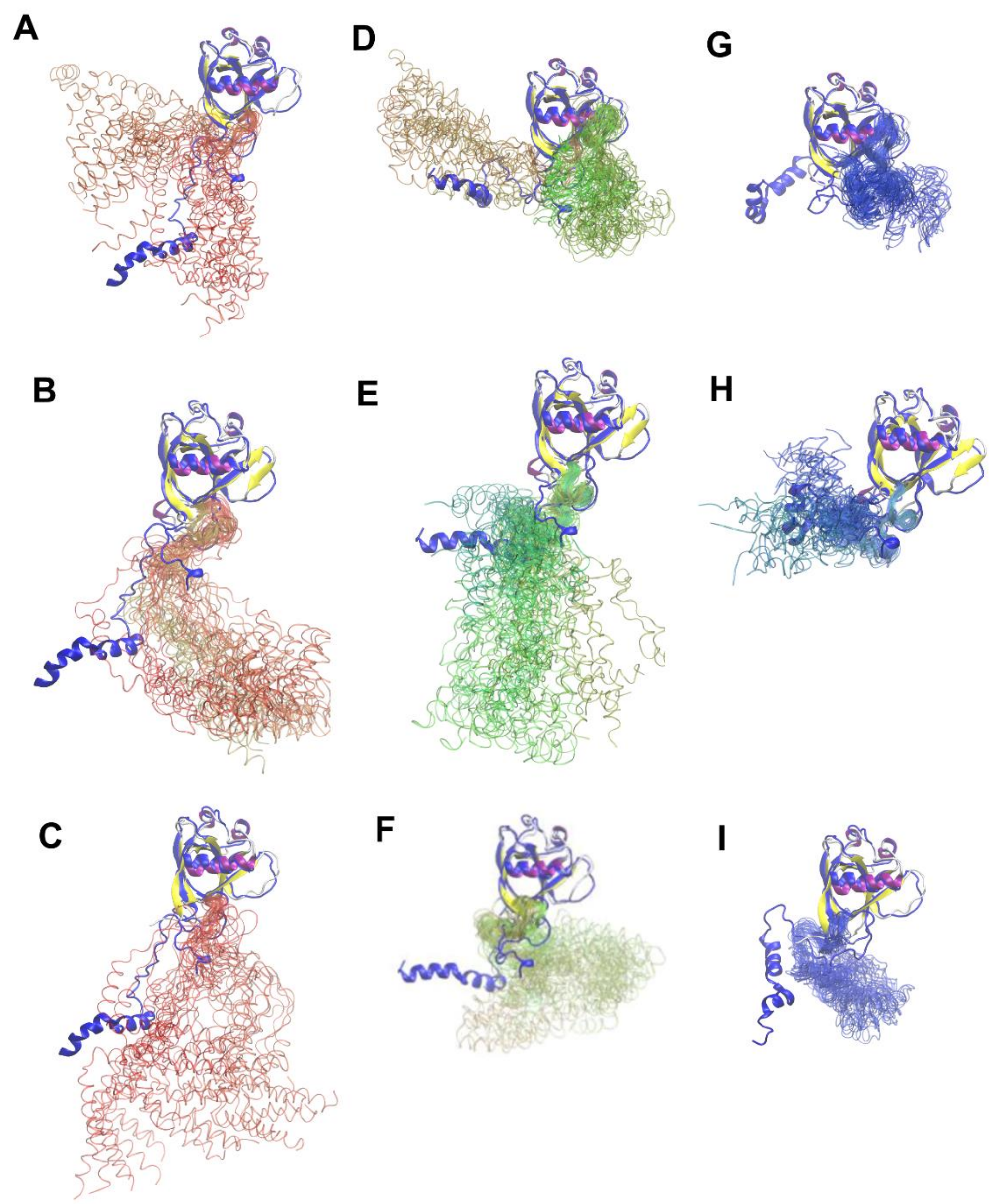

Figure S4. Time lapse representation for trajectories obtained from replicas 2 (A-C), 3 **(D-F)** and 4 **(G-I)**. For each panel 25 equally spaced frames ranging the specified time range are depicted. Residues 1 to 128 are represented as thick ribbons and colored by secondary structure (purple for α-helix and yellow for β-strands), and residues 129 to 180 are represented as thin ribbons and RGB colored from the beginning to the end of simulation time, respectively. In each panel, the closest conformation from the models obtained with MultiFoXS (thick blue ribbons) was superimposed choosing for the alignment the backbone α-carbon atoms ranging residues 15 to 120.

Figure S5

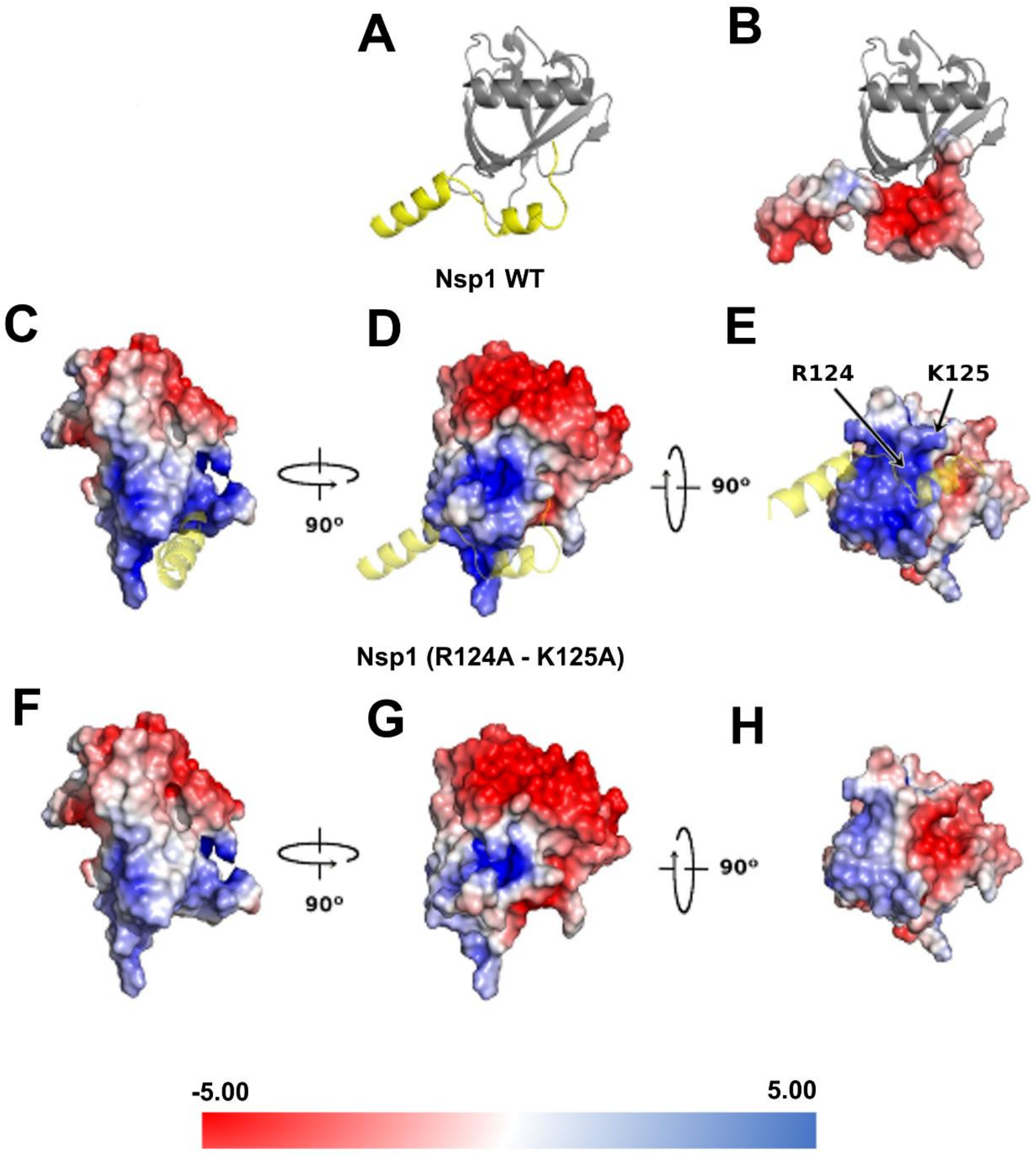

Figure S5. **Nsp1 $^{\text{SARS-CoV2}}$ electrostatic potential.** (**A)** Cartoon representation for the structure of the complex between N-terminal (NTD, grey) and C-terminal (CTD, yellow) Nsp1 $^{\text{SARS-CoV2}}$ domains obtained using HPEPDOCK for peptide-protein docking. **(B)** APBS calculation for C-terminal domain, mapped over a surface representation in the best pose obtained from docking result. (**C-E)** APBS calculation for Nsp1 $^{\text{SARS-CoV2}}$ NTD mapped over a surface representation and presented from 3 different angles, where panel C can be named as "side view", panel D as "front view", and panel E as "bottom view". Nsp1 $^{\text{SARS-CoV2}}$ CTD is depicted as yellow transparent ribbons. **(F-H)** APBS calculation for the double variant R124A/K125A Nsp1 $^{\text{SARS-CoV2}}$, represented analogously as for the *wild type* Nsp1 $^{\text{SARS-CoV2}}$ in panels **C** to **E**.

Figure S6

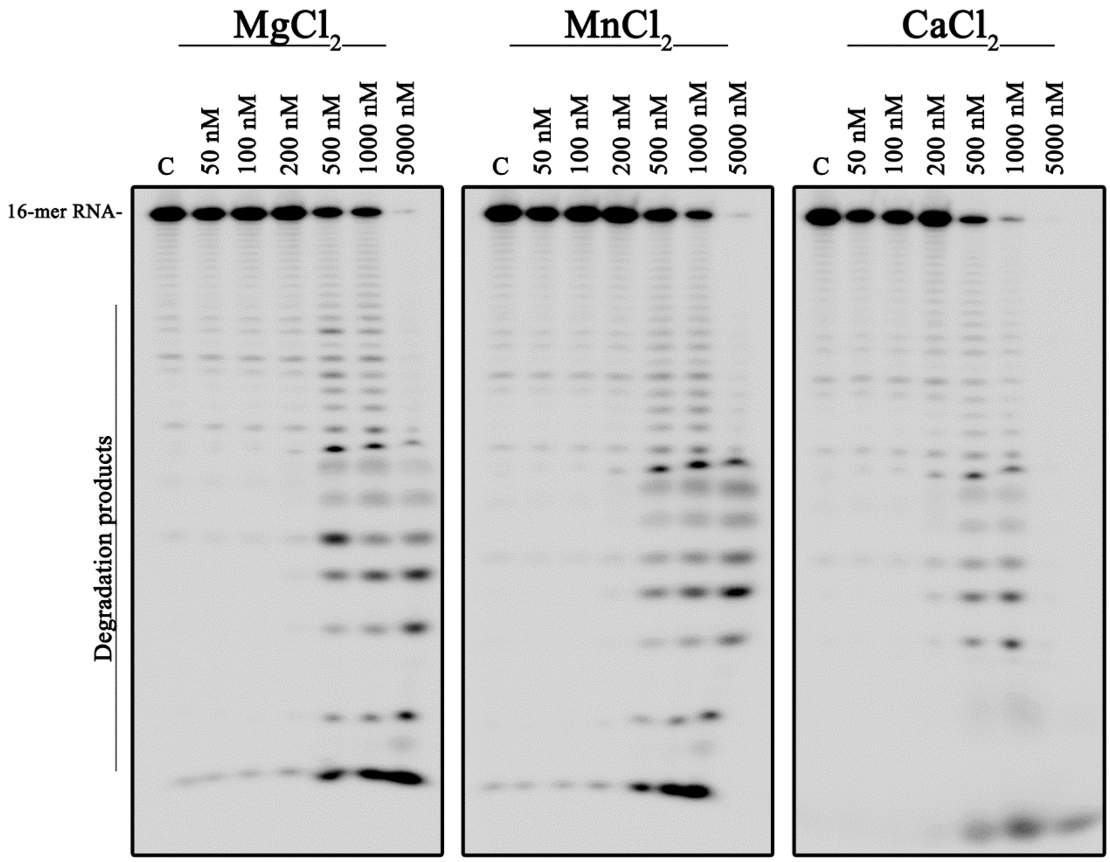

Figure S6. RNA nuclease activity of Nsp1 $^{SARS\text{-}CoV2}$. Nsp1$^{SARS\text{-}CoV2}$ (0.05 - 5 µM) was incubated with 50 nM of 16-mer RNA in the presence of 10 mM of $MgCl_2$, $MnCl_2$, or $CaCl_2$ for 60 min. Reactions were analyzed on 7 M urea/20% polyacrylamide gels. *C*, control reactions in the absence of Nsp1$^{SARS\text{-}CoV2}$.

Figure S7

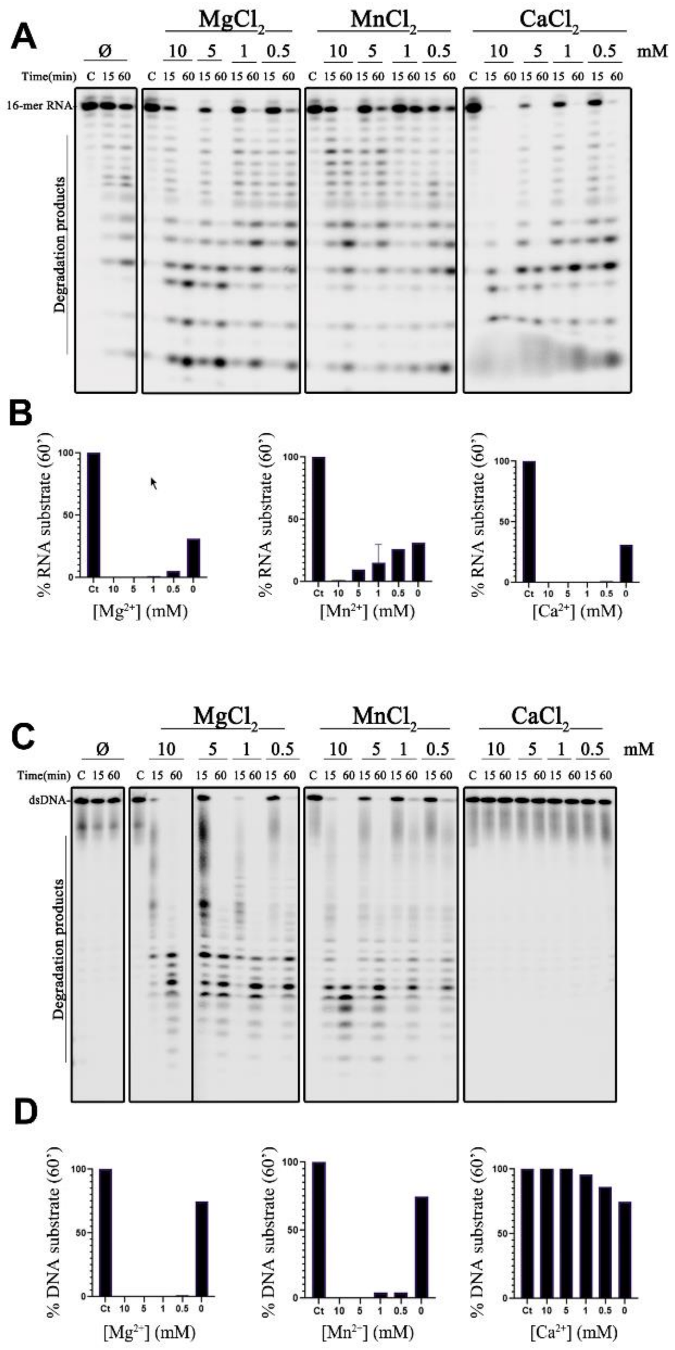

Figure S7. Metal cofactor-dependent activity of Nsp1 $^{\text{SARS-CoV2}}$. (A,B) 5 µM of Nsp1 $^{\text{SARS-CoV2}}$ was incubated with 50 nM of 16-mer RNA in the presence of different concentrations of $MgCl_2$, $MnCl_2$, and $CaCl_2$ (0.5 to 10 mM). Reactions were analyzed on 7 M urea/20% polyacrylamide gels. Ø, reaction in the absence of divalent ions; *C*, control reactions in the absence of Nsp1 $^{\text{SARS-CoV2}}$; time points are indicated in the top of each panel. **(C,D)** 5 µM of Nsp1 $^{\text{SARS-CoV2}}$ was incubated with 50 nM of dsDNA in the presence of different concentrations of $MgCl_2$, $MnCl_2$, and $CaCl_2$ (0.5 to 10 mM). Reactions were analyzed on 7 M urea/20% polyacrylamide gels. Ø, reaction in the absence of divalent ions; C, control reactions in the absence of Nsp1 $^{\text{SARS-CoV2}}$; time points are indicated in the top of each panel.

Figure S8

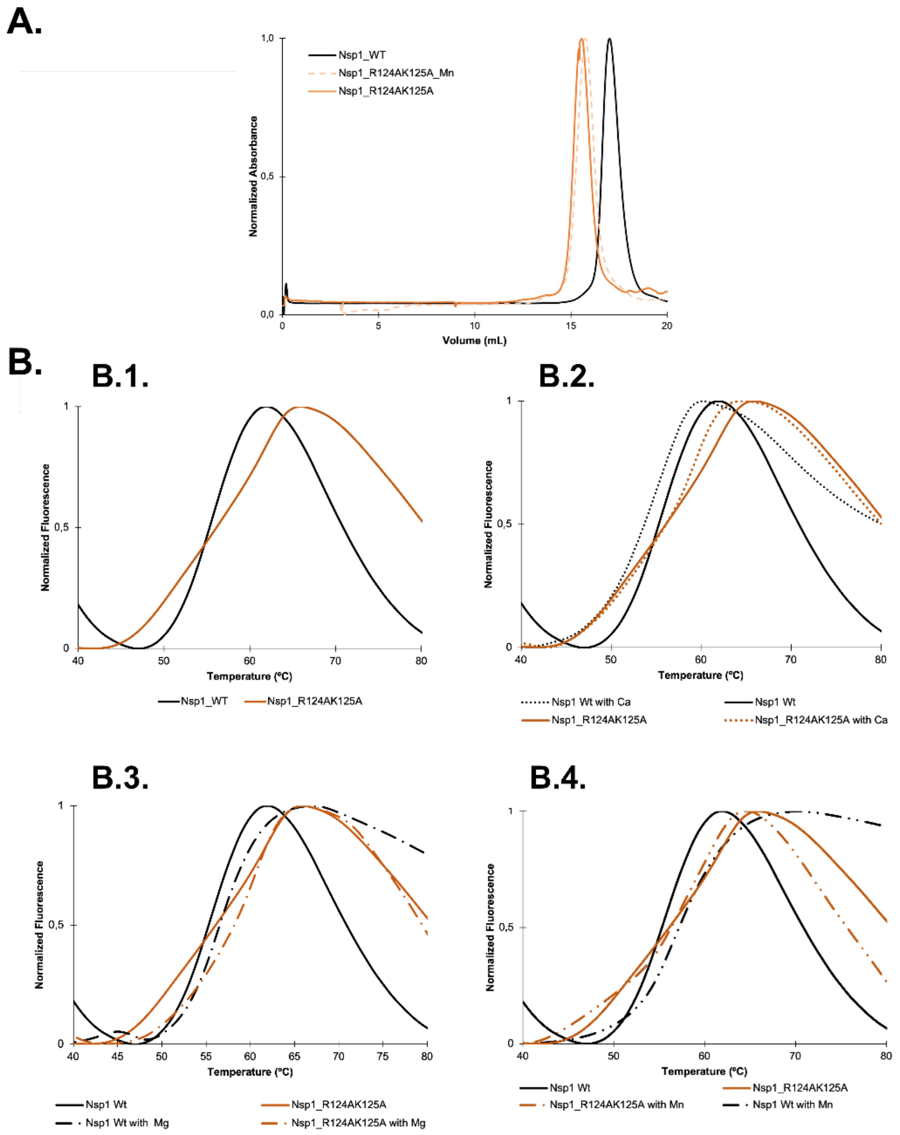

Figure S8. Size exclusion chromatography (SEC) and Thermal shift assay (TSA) of the double variant R124A/K125A Nsp1$^{SARS\text{-}CoV2}$. (A) SEC of Nsp1 $^{SARS\text{-}CoV2}$ without metal control (black line) and the double variant R124A/K125A Nsp1$^{SARS\text{-}CoV2}$ without metal (orange line) and double variant R124A/K125A Nsp1$^{SARS\text{-}CoV2}$ with 50mM of $MnCl_2$ (orange line ---). **(B)** ***B₁.*** TSA of Nsp1 $^{SARS\text{-}CoV2}$ (black line) and the double variant R124A/K125A Nsp1$^{SARS\text{-}CoV2}$ (orange line) without metal. ***B_2.*** TSA of Nsp1 $^{SARS\text{-}CoV2}$ (control, black line) and in the presence of 50 mM $CaCl_2$ (….), and the double variant R124A/K125A Nsp1$^{SARS\text{-}CoV2}$ (orange line) and with 50 mM $CaCl_2$ (….). ***B_3.*** TSA of Nsp1 $^{SARS\text{-}CoV2}$ (control, black line) and in the presence of 50 mM $MgCl_2$ (-.-.), and the double variant R124A/K125A Nsp1$^{SARS\text{-}CoV2}$ (orange line) and with 50 mM $MgCl_2$ (-.-.). ***B_4.*** TSA of Nsp1 $^{SARS\text{-}CoV2}$ (control, black line) and in the presence of 50 mM $MnCl_2$ (-..-..), and the double variant R124A/K125A Nsp1$^{SARS\text{-}CoV2}$ (orange line) and with 50 mM $MnCl_2$ (-..-..).

Figure S9

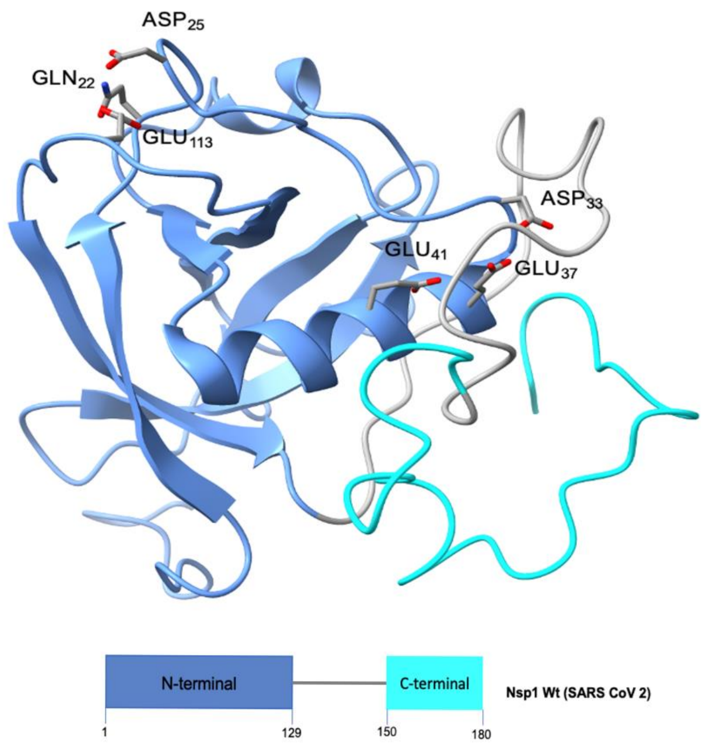

Figure S9. Structure model of Nsp1 SARS-CoV2 (PDB:8AOU) highlighting the amino acid residues predicted for the metal binding. The NTD is represented in blue, the CTD is cyan and the connecting loop in gray. Amino acid residues were predicted for metal binding using IonCom server are illustrated with sticks.

Table S1. SAXS data of Nsp1$^{SARS\text{-}CoV2}$ interactions at 25°C.

Data collection parameters	
Instrument:	ESRF BM29
Wavelength (Å)	0.99
q-range (Å^{-1})	0.007-0.5
Sample-to-detector distance (m)	2.81
Concentration range (mg/mL)	1-6
Temperature (K)	293
Detector	Pilatus P3-2M
Flux (photons/s)	$1.4*10^{12}/1*10^{13}$
Beam size at sample (μm)	500*200

Structural parameters (HPLC)	
I_0 (kDa) [from Guinier]	17.1
R_g (Å) [from Guinier]	22.64 ± 0.52
R_g (Å) [from P(r)]	21.91
qmin R_g – qmax R_g used for Guinier (Å^{-1})	$2.8*10^{-2}$-$5.7*10^{-2}$
Volume (Å^3)	$4.5*10^4$
D_{max} (Å) (Moore L2-NORM)	78
Calculated theoretical MW (kDa):	19.75
Calculated MW (kDa):	24

software employed	
Primary data reduction:	FreeSAXS, BM29 autoprocessing pipeline
Data processing	ScatterIV, BioXTAS RAW and multiFoXS

Table S2. Radius of gyration (Rg) and fit score (χ^2) obtained for the 5 predictions obtained with MultiFoXS, as a function of the number of states used in each model.

**Number of states**	χ^2	**Rg (Å)**
1	2.29 ± 5.07	22.16
2	1.77 ± 4.77	20.78; 28.05
3	1.74 ± 1.14	22.48; 19.51; 28.05
4	1.73 ± 0.11	22.16; 20.78; 19.05; 28.05
5	1.73 ± 0.01	22.16; 22.48; 21.61; 19.05; 28.05
